# Social media and internet search data to inform drug utilization: A systematic scoping review

**DOI:** 10.3389/fdgth.2023.1074961

**Published:** 2023-03-20

**Authors:** Roman Keller, Alessandra Spanu, Milo Alan Puhan, Antoine Flahault, Christian Lovis, Margot Mütsch, Raphaelle Beau-Lejdstrom

**Affiliations:** ^1^ Epidemiology, Biostatistics and Prevention Institute, University of Zurich, Zurich, Switzerland; ^2^ Future Health Technologies, Singapore-ETH Centre, Campus for Research Excellence and Technological Enterprise (CREATE), Singapore, Singapore; ^3^ Saw Swee Hock School of Public Health, National University of Singapore, Singapore, Singapore; ^4^ Institute of Global Health, University of Geneva, Geneva, Switzerland; ^5^ Division of Medical Information Sciences, University Hospitals of Geneva, Geneva, Switzerland; ^6^ Department of Radiology and Medical Informatics, Faculty of Medicine, University of Geneva, Geneva, Switzerland

**Keywords:** surveillance, social media, drug utilization, systematic scoping review, user-generated data, internet search, Google trends

## Abstract

**Introduction:**

Drug utilization is currently assessed through traditional data sources such as big electronic medical records (EMRs) databases, surveys, and medication sales. Social media and internet data have been reported to provide more accessible and more timely access to medications' utilization.

**Objective:**

This review aims at providing evidence comparing web data on drug utilization to other sources before the COVID-19 pandemic.

**Methods:**

We searched Medline, EMBASE, Web of Science, and Scopus until November 25th, 2019, using a predefined search strategy. Two independent reviewers conducted screening and data extraction.

**Results:**

Of 6,563 (64%) deduplicated publications retrieved, 14 (0.2%) were included. All studies showed positive associations between drug utilization information from web and comparison data using very different methods. A total of nine (64%) studies found positive linear correlations in drug utilization between web and comparison data. Five studies reported association using other methods: One study reported similar drug popularity rankings using both data sources. Two studies developed prediction models for future drug consumption, including both web and comparison data, and two studies conducted ecological analyses but did not quantitatively compare data sources. According to the STROBE, RECORD, and RECORD-PE checklists, overall reporting quality was mediocre. Many items were left blank as they were out of scope for the type of study investigated.

**Conclusion:**

Our results demonstrate the potential of web data for assessing drug utilization, although the field is still in a nascent period of investigation. Ultimately, social media and internet search data could be used to get a quick preliminary quantification of drug use in real time. Additional studies on the topic should use more standardized methodologies on different sets of drugs in order to confirm these findings. In addition, currently available checklists for study quality of reporting would need to be adapted to these new sources of scientific information.

## 1. Introduction

Drug utilization research has been defined as “an eclectic collection of descriptive and analytical methods for the quantification, the understanding and the evaluation of the processes of prescribing, dispensing and consumption of medicines, and for the testing of interventions to enhance the quality of these processes.” (1). Accurate and timely estimates of pharmaceutical drug utilization patterns are considered critical for assessing drug safety, effectiveness, access to drugs, and patients' care (2, 3). Higher than expected use of some medications in a specific country (e.g., opioids in the United States) should be flagged rapidly as it could point to potential drug abuse). Timely assessment of drug utilization could be used to investigate the effectiveness and safety of medications for this new disease (4). On the contrary, when detected early, suboptimal use of essential medicines or vaccines could trigger health policymaking to prevent the resurgence of preventable morbidity.

Traditional ways to retrieve data on the use of drugs based on surveys, prescription rates, and drug sales tend to be slow, expensive, difficult to obtain, limited in geographic scope, and may not accurately capture a representative sample of the population. Currently, accessing the appropriate databases and analyzing drug utilization can take up to a year (sometimes even more). These limitations in retrieving drug utilization data can affect the health of populations.

In the last decade, web data such as social media and internet search data have been shown to be useful for infectious disease surveillance. In 2009, a study based on Google Flu Trends showed that worldwide influenza virus activity could be monitored using the Google search engine (5). It was found that the frequency of influenza-associated search terms highly correlated with the number of physician visits for influenza-like symptoms (5). Similar approaches have also been used in pharmacovigilance-focused studies, which deal with detecting, comprehending, and preventing adverse drug events (6, 7). Similarly, the potential of using social media data to detect adverse drug reactions (8) as well as its use for infectious disease surveillance (9–11) have been recognized in the literature, and an increasing number of studies utilize web data to assess drug utilization (12–14).

Therefore, studies on web data could provide evidence of a complementary way to access information on drug utilization compared to traditional methods. We conducted a systematic scoping review and aimed to assess the content and quality of existing research using social media and internet search data to study drug utilization volumes compared to other sources of drug utilization information. This review was performed before the start of the COVID-19 pandemic as we believe that the specific media attention on some medications during this period may not reflect the association that could be made between drug web data and drug utilization in more usual circumstances.

## 2. Methods

### 2.1. Reporting standards

We performed a systematic scoping review and followed the Preferred Reporting Items for Systematic Reviews and Meta-Analyses extension for Scoping Reviews (PRISMA-ScR) checklist (15) (Supplementary File S5). The review protocol is available in the online Supplementary Material (File S1).

### 2.2. Search strategy

A literature search was conducted in September 2016, updated in November 2019, and included PubMed Medline, EMBASE, Scopus, and Web of Science. The search strategy was developed including an experienced pharmacoepidemiologist and counseling by an information specialist. The PubMed Medline search strategy is available in the online Supplementary Material (File S2).

### 2.3. Selection criteria

We included studies if they: (1) were primary research studies that involved web data including social media or search engine data such as Google Trends, Google Correlate, Google Insights for Search, Google search engine, Facebook, Twitter, and Instagram; (2) involved any kind of comparison data such as drug sales or drug prescription volumes acquired from surveys, registry data, physician databases, and others. Not all of these data originated from validated sources; and (3) included any kind of drug utilization data such as utilization frequencies of vaccines, vitamins, supplements, nicotine alternatives, prescription drugs, and over-the-counter drugs for both data sources.

Articles were excluded if they: (1) focused on E-cigarettes; (2) involved incidence rates of diseases instead of drug utilization volumes; or (3) involved only web data sources but no other kind of comparison data source.

In addition, we excluded non-English study documents, literature reviews, posters, PowerPoint presentations, articles presented at doctoral colloquia, or if the article's full text was not accessible to the study authors (e.g., conference abstracts). Only peer-reviewed proceedings were included in this review.

### 2.4. Selection process

All identified references were downloaded into Endnote, where duplicates were removed. Two independent reviewers conducted the screening with the free online tool Cadima (15). First, titles and abstracts were screened, followed by screening of the articles' full texts. The reference lists of the included articles were checked for additional studies. Any remaining disagreements about study inclusion or exclusion were resolved by a third investigator.

### 2.5. Data extraction

One reviewer independently extracted the prespecified information of the articles into a Microsoft Excel sheet with 22 columns containing information on the following aspects: (1) General information on the included studies (e.g., study objective), (2) characteristics of the involved data sources (e.g., web data source), and (3) additional study items (e.g., conflict of interest). The full list can be accessed in the online Supplementary Material (File S4).

Additionally, the reporting quality of the included studies was assessed using the STROBE checklist (16) (Strengthening the Reporting of Observational Studies in Epidemiology) as well as the statement's extensions RECORD (Reporting of studies conducted using observational routinely collected data) (17) and RECORD-PE (Reporting of studies conducted using observational routinely collected data for pharmacoepidemiological research) (18). Items were excluded if they were considered out of scope for the investigated population of research studies. One reviewer subsequently reviewed the adherence of the articles to the checklists' items. The checklist items were marked “yes” if the item was described satisfactorily well, “partly” if described partially, and “no” if it was not described at all. If an item was not applicable due to a study's nature or design, the item was marked “n/a”.

One reviewer additionally reviewed the study authors' perceptions of the challenges of using web data for drug utilization estimation reported in the discussion sections of the papers. The abstracted data items were verified by a second reviewer, and any disagreements were resolved in consensus. The full list can be accessed in the online Supplementary Material (File S3). The extracted data were synthesized narratively. Descriptive statistics were performed using Microsoft Excel (e.g., frequencies, and measures of central tendency).

### 2.6. Risk of bias assessment

Risk of bias assessment was not conducted, which is consistent with the scoping review methods manual by the Joanna Briggs Institute (19).

## 3. Results

### 3.1. Study flow

**Figure 1:**
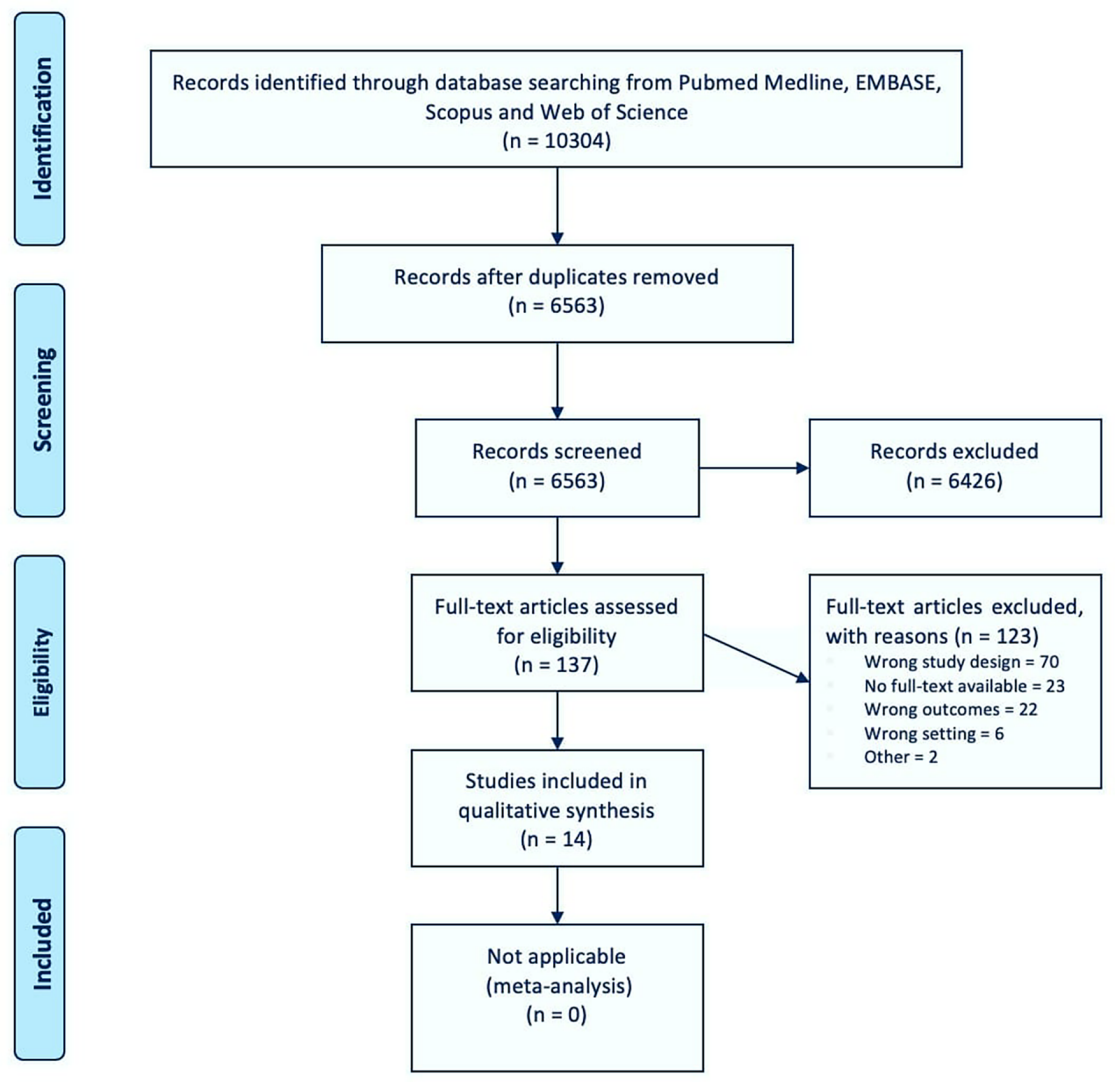
PRISMA flow diagram of included studies.

A total of 6,563 deduplicated citations from electronic databases were screened (Figure 1). Of these, 6,427 (98%) papers were excluded during the title- and abstract-screening process, leaving 137 (2%) articles eligible for full-text screening. A total of 123 (90%) full texts were found to be ineligible for study inclusion, the most common reason being wrong study design as they did not include relevant datasources or any comparison with drug utilization data [see exclusion criteria 2, *n* = 70 (57%)]. Ultimately, 14 (10%) papers were considered eligible for inclusion. A first search was conducted in September 2016, identifying eight eligible articles, and the updated search in November 2019 yielded six additional papers. The full list of included documents can be found in the online Supplementary Material (File S4).

### 3.2. Characteristics of included studies

The articles' publication dates ranged from 2010 to 2019, with 93% (13/14) of papers published from 2014 onwards (Table 1). The document types comprised journal articles (79%) and (full) conference papers (21%) (see Supplementary File S4).

**Table 1:** Characteristics of included studies (ordered by year of publication).

Reference (country^1^)	Study design	Objective	Web data source (country), *data measure*	Comparison data source (country), data measure	Statistics of comparison	Medication of interest	Main findings	Main findings
Schuster 2010 (20) (United States)	Retrospective longitudinal study	To evaluate the association of the relative search volume of the search term Lipitor with Lipitor global revenues.	Google Trends/Google Insight for Search (United States), Mean number of Google queries,	Pfizer Annual Shareholder Reports 2004–2008 (worldwide), Global revenues,	Quantitative, Pearson correlation coefficient	Lipitor (atorvastatin calcium), simvastatin (Zocor)	- The percentage change in annual Lipitor global revenues decreased from 2004 to 2008 and significantly correlated with the mean Google query index for Lipitor.	
Simmering 2014 (21) (United States)	Retrospective longitudinal study	To evaluate the association between drug utilization estimates of several seasonal prescription drugs and the corresponding Google Trends search volume.	Google Trends, search volume	Medical Expenditure Panel Survey (MEPS) (United States), Nationally representative drug utilization estimates	Quantitative, Cross-correlation function	Amoxicillin, azelastine, azithromycin, benzonatate, cefdinir, ciprofloxacin, levofloxacin, moxifloxacin, olopatadine.	- Only three out of nine seasonal drugs had enough outpatient dispensing events in the MEPS data to construct a time series suitable for analysis (amoxicillin, azithromycin and cefdinir). These three drugs showed positive correlation between the search volume and drug utilization estimates at lags near 0 (i.e. in the same week) and at year intervals as well as a strong negative relationship at half-years intervals.	
Skeldon 2014 (22) (Canada)	Ecologic analysis	To evaluate the association of two direct-to-consumer advertising (DTCA) campaigns with the volume of Internet searches for “Avodart” (dutasteride) and “Flomax” (tamsulosin) and to evaluate the association of the DTCA campaigns with the prescription rates of dutasteride and tamsulosin.	Google trends (United States), search term frequency	IMS Health (United States), Prescription rates	No direct comparison but parallel reporting of results of both data sources	Dutasteride (Avodart®), tamsulosin (Flomax®)	- The dutasteride campaign was significantly correlated with increases in search volumes for Avodart® and Flomax® and with increases in the prescription of dutasteride and tamsulosin. - The tamsulosin campaign was significantly associated with increased Flomax® search volumes and with immediate increases in the prescription of dutasteride and tamsulosin.	
Gahr 2015 (23) (Germany)	Retrospective longitudinal study	To evaluate the association of annual prescription volumes of several antidepressants with marketing approval in Germany with corresponding Google Trends web search query volumes.	Google Trends (Germany), search term frequency	Drug prescription report (Germany), Prescription volumes at the expense of the statutory health insurance	Quantitative, Pearson's r (Interpreted by the review authors and not Person's r as stated in the paper.)	agomelatine, bupropion, citalopram, escitalopra, fluoxetine, fluvoxamine, paroxetine, sertraline	Significant correlations between substance-specific annual prescription volumes and corresponding annual query volumes found for each substance: (agomelatine: r 0.968, R^2^ 0.932; bupropion: r 0.962, R^2^0.925, citalopram: r 0.970, R^2^ 0.941, escitalopram: r 0.824, R^2^ 0.682, fluoxetine: r 0.885, R^2^ 0.783, paroxetine: r 0.801, R^2^ 0.641, sertraline: r 0.880, R^2^ 0.689; *p* 0.01 for all correlations).	
Jha 2015 (24) (United States)	Ecological analysis	To investigate trends in media reports and public interest of bisphosphonates using Google Trends as well as to estimate the trends in oral bisphosphonate use among patients aged ≥55 years using national health survey data.	Google Trends (United States), search term frequency	The Medical Expenditure Panel Survey (MEPS) (United States), Estimation of medication utilization based on prescription volumes	No direct comparison but parallel reporting of results of both data sources	Oral bisphosphonates	- The prevalence of oral bisphosphonate use declined by greater than 50% between 2008 and 2012 (*p* < 0.001) after increasing use for more than a decade.	- A series of spikes in search volume for Fosamax® (alendronate) occurred between 2006 and 2010 immediately following media reports of safety concerns.
Kalichman 2015 (25) (United States)	Retrospective longitudinal study with cross-sectional comparison	To examine the associations of the internet search activity for H1N1 and human papilloma virus (HPV) disease and vaccine information with H1N1 and HPV vaccine uptake.	Google Insight for Search (United States), search term frequency	Centers for Disease Control and Prevention (CDC) (United States), Vaccination coverage	Quantitative, Spearman's rho correlation and ordinal regression analysis for multivariable models	H1N1 flu vaccine, HPV vaccine	- The search terms *H1N1* and *vaccine* significantly correlated with H1H1 vaccine uptake, with rho ranging from 0.45 to 0.57 for *H1N1* and from 0.32 to 0.49 for *vaccine* (except for persons older than 65 years: rho = 0.22, *p* > 0.05). Ordinal regression showed that the *H1N1* search term was independently associated with H1N1 vaccine uptake (Wald *χ*^2^ = 10.41, *p* < 0.001). - Similar results for the correlation between the search volume of the term *vaccine* and HPV vaccine uptake	
Crowson 2016 (26) (United States)	Retrospective longitudinal study	To evaluate common ototopical antibiotics’ prescription volumes association with corresponding Google Trends search volumes and to investigate the seasonality of national prescription volumes and Google Trends search volume.	Google Trends (United States), search term frequency	Medicaid (United States), Prescription volumes	Quantitative, Pearson's correlation coefficient	Ciprofloxacin-dexamethasone,’’ ““Cortisporin,”’ ‘‘Ofloxacin,’’	- Google Trends showed significant correlations to Medicaid prescription volumes for Ciprofloxacin-dexamethasone (r = 0.38, *p* = 0.046), Ofloxacin (r = 0.74, *p* < 0.001), Cortisporin (r = 0.49, *p* = 0.008)	- Google Trends showed sinusoidal seasonality similar to Medicaid prescription data with annual peaks in the summer (June to September).
Hansen 2016 (27) (Denmark)	Proof-of-concept of prediction models	To develop and evaluate prediction models using clinical and web-mined data for predictions about future vaccination uptake for all official recommended children Vaccines in Denmark.	Google Trends (Denmark), search query frequency	State Serum Institut (Denmark), Vaccine uptake	Quantitative, root mean squared error	All official children vaccines in Denmark: DiTeKiPol-1 to 4, PCV-1 to 3, MMR-1, MMR-2 (4), MR-2 (12), HPV-1 to 3	- For 10/13 recommended childhood vaccines in Denmark the developed machine learning method combining web and clinical data for prediction outperformed predictions using either clinical or web data alone. - Using only web data gives predictions with an overall error slightly worse than for using clinical data only.	
Jankowski 2016 (28) (Poland)	Retrospective longitudinal study cross-sectional comparison	To develop a method using Google search engine data to rank psychoactive drugs according to their popularity and to qualitatively compare the popularity ranking to international drug report data.	Google search engine, frequency of website hits	UNODC World Drug Report 2011 (worldwide) and European Drug Report 2014: Trends and Developments (European Union, Turkey, Norway), Number of drug seizures	Qualitative, Popularity ranking list	Alcohol, amphetamine, benzodiazepines, buprenorphine, butane, cannabis, cocaine, ecstasy, GHB, heroin, ketamine, khat, LSD, mephedrone, methadone, methamphetamine	- Alcohol was found to be the most popular psychoactive drug with a relative popularity index of 100%, followed by cannabis with 15.2% and with cocaine (15.1%).	- The drug popularity ranking was quite similar to the UNODC report data of 2011 as well as the European Drug Report 2014: Trends and Developments.
Song 2017 (29) (United States)	Retrospective longitudinal study	To develop a method using Twitter data for flu vaccination monitoring and to evaluate the method against official flu vaccination surveillance data.	Twitter (United States), number of twitter posts	Flu vaccination rate surveillance system used by the U.S. DHHS (United States), Immunization rates of flu vaccination	Quantitative, Pearson correlation coefficient	Influenza (flu) vaccination	Correlation coefficients between 0.876 and 0.997 and *p*-values of less than 0.00001 indicate a significant, positive linear relationship between the number of twitter posts and flu vaccination immunization rates.	
Hansen 2018 (30) (Denmark)	Proof-of-concept of prediction models	To develop and evaluate prediction models using web search and antimicrobial purchase data for predictions about future antimicrobial drug consumption.	Google Health Trends (Denmark), search query frequency	Register of Medicinal Product Statistics (Denmark), Sales of antimicrobials	Quantitative (prediction models were quantitative, qualitative comparison), root mean squared error and mean absolute error	Antibiotics; subgroup: beta-lactamase sensitive penicillins (J01CE)	- Overall, the use of web data only gives predictions that are slightly more erroneous, but generally not that far off, from those made when using only historical antimicrobial purchase data. - Best predictions when combining both web search and antimicrobial purchase data.	
Huang 2018 (31) (United States)	Cross-sectional study	To develop a method based on a machine learning classifier that employs Twitter data for real-time influenza vaccination surveillance and to evaluate the method by comparing to published government survey data.	Twitter (United States), number of twitter posts	Centers for Disease Control and Prevention ‘s (CDC) FluVaxView system (United States), Influenza vaccination activity data	Quantitative, Pearson correlation coefficient	Influenza vaccination	- Strong correlations of 0.799 (95%CI: 0.797 to 0.801) between monthly Twitter estimates and governmental data, with geographical correlations of 0.387 (95%CI: 0.362 to 0.394) at US state level and of 0.467 (95%CI: 0.445 to 0.483) at the regional level. - More tweets were found for female twitter users compared to males, consistent with the CDC results on vaccine uptake.	
Kamiński 2019 (32) (Poland)	Retrospective longitudinal study with cross-sectional comparison	To analyse the association of the Google Trends’ relative search volume for the topics antibiotics and probiotics with antibiotic consumption worldwide.	Google Trends (worldwide), relative search volume	The Center for Disease Dynamics Economics & Policy (worldwide), Antibiotic consumption	Quantitative, Spearman rank-correlation	Antibiotics, probiotics	Antibiotic consumption was significantly associated with the relative search volume of probiotics (Rs = 0.35; *p* < 0.01), but not antibiotics (Rs = 0.14; *p* > 0.05).	
Mimura 2019 (33) (Japan)	Retrospective Observational Study	To examine prescription trends in heparinoid (moisturizer) use and analyse their association with Google Trends search volume.	Google trends (Japan), search term frequency	Administrative claims database provided by JMDC Inc (Japan), Prescription volume	Quantitative, Cross-correlation	Heparinoid	- Internet searches were significantly correlated with total heparinoid prescription at 0-months lag (correlation coefficient = 0.25, *P* = 0.005). - Internet searches were significantly correlated with heparinoid prescription among ages 20-59 years at –1-month lag in Google Trends (correlation coefficient = 0.30, *P* = 0.001).	

^1^ Country indicated for the corresponding author.

### 3.3. Data source characteristics

Of all reviewed articles, the most employed web data source was Google Trends' search volumes assessed in eight (57%) studies (21–24, 26, 27, 32, 33). Two (14%) studies used Twitter posts (22, 34), and two (14%) other studies utilized search volumes from former Google services similar to Google Trends: specifically, the Google Health Trends API (30) and Google Insights for Search (25). One (7%) study utilized both Google Insights for Search' and Google Trends' search volume (20), and another (7%) study assessed the frequency of website hits where a certain keyword is found using the Google search engine (28).

Datasources used for comparison with Web data included: Elven (79%) studies used data from public/government organizations drug utilization estimates as comparator to the web data (21, 24, 25, 27, 29, 31, 32). U.S. databases [Medical Expenditure Panel Survey (MEPS) (21, 24), Database from Centers for Disease Control and Prevention (CDC) (25, 31), Center for Disease Dynamics Economics & Policy (32), the flu vaccination rate surveillance system used by the U.S. Department of Health and Human Services (DHHS) (29), Medicaid (26), State Serum Institute (27), Register of Medicinal Product Statistics (30), Drug prescription report, Germany (23), European Drug Report 2014: Trends and Developments (28), UNODC World Drug Report 2011 (28)], and three studies (21%) used privately owned databases [the 2004 to 2008 Pfizer Annual Shareholder Reports (20), IMS Health (22) and the administrative claims database provided by JMDC Inc. (33)].

Twelve (86%) out of fourteen studies provided the time of data collection for both the web and the comparison data source. In these studies, the web data were gathered for a median duration of 5.3 years (interquartile range of 3.9 to 8.6 years), while the comparative data were collected for a median duration of 5.0 years (interquartile range of 3.7 to 9.6 years). One (7%) study only reported the time of data collection for the comparison data source (21), while in another (7%) study, the time of data collection could not conclusively be identified (28).

### 3.4. Approaches used for comparisons

Nine (64%) of the fourteen studies quantitatively compared web-mined and comparison data using different types of correlation analyses (Pearson -, Spearman - and Cross-correlation) (20, 21, 23, 25, 26, 29, 31–33). Two studies (14%) quantitatively compared the performance of different prediction models (27, 30) using web and comparison data in terms of root mean squared and mean absolute error. One study qualitatively compared different popularity ranking lists (28). Furthermore, two (14%) studies did not directly compare drug utilization volumes but reported the results of both data sources as part of an ecological analysis without statistical comparison (22, 24).

### 3.5. Therapeutic classes of drugs assessed

With a total of four (28%) studies, vaccines were the most frequently investigated drug class (25, 27, 29, 31). Two (14%) studies examined antibiotics (26, 30), and one (7%) study focused on both antibiotics and probiotics (32). The remaining studies included: Psychoactive drugs (28), statins (20), drugs for benign prostatic hyperplasia (22), antidepressants (23), medications with seasonal patterns (21), moisturizer (heparinoid) (33) and oral bisphosphonates (24).

### 3.6. Main findings

Overall, positive associations between drug utilization estimates reported in web data sources and comparison data sources were found in all studies, with significant results reported in eight of the nine studies that used correlation analyses (20, 21, 23, 25, 26, 29, 31, 33). Kamiński et al. found antibiotic consumption to be significantly associated with internet search data of probiotics but not antibiotics (32). Kalichman et al. found that the internet search term *H1N1* independently predicted H1N1 vaccine coverage, while the search term *vaccine* independently predicted HPV vaccination coverage as results of ordinal regression analyses (25). Two studies built and evaluated models to predict future drug utilization and reported the best predictions when combining web and comparison data (27, 30). Jankowski et al. developed a drug popularity ranking list using internet search data and found the list to be similar to those reported by two international drug data sources (28). Two studies conducted ecological analyses (22, 24), of which Skeldon et al. study reported both increased web search interest and drug prescription rates, separately after two sequential advertising campaigns (22). The study of Jha et al. found a series of temporally correlated spikes in internet search activity and a decline in drug utilization estimates following media reports of medication safety concerns (24).

Three studies found similar seasonal patterns across the web and comparison data sources (21, 26, 31). Moreover, one study found correlations between internet search volumes and drug prescription volumes not only at the same time but also following a one-month time lag for the population aged 20 to 59 years, suggesting that people obtain health-related information from the internet, which may subsequently affect their behavior and medication requests (33).

### 3.7. Assessment of the reporting quality

The adherence of the articles to the individual items of the STROBE, RECORD, and RECORD-PE statements is presented in Table 2. In over 80% of the studies, the following items were reported: title and abstract (1.1), background rationale (2), objectives (3), variables (7.1.b), statistical methods (12-a), and outcome data (15). The following (sub-)items were considered in more than 20 to 50% of the studies: title and abstract (1-a, 1-b, 1.2), study design (4), setting (5), data access (12.1), key results (18), limitations (19.1), interpretation (20), generalisability (21), and funding aspects. Less than 20% of the studies described the following items: variables (7.1, 7.1-a), bias (9), statistical methods (12-e), participants (13-c), other analyses (17), and accessibility of protocol, raw data, and programming code (22.1).

**Table 2:** Reporting of items of the STROBE statement (strengthening the reporting of observational studies in epidemiology) complemented with items from the RECORD and RECORD-PE checklists [reporting of studies conducted using observational routinely collected data (RECORD) and RECORD statement for pharmacoepidemiological research (RECORD-PE)].

Item		Category	Item description	Total coverage							
				Yes (%)		Partly (%)		No (%)		Not applicable (%)	
		Title and abstract								
1	(a)		Indicate the study's design with a commonly used term in the title or the abstract.	4	(29)	1	(7)	9	(64)	0	(0)
	(b)		Provide in the abstract an informative and balanced summary of what was done and what was found.	11	(79)	3	(21)	0	(0)	0	(0)
	1.1*		The type of data used should be specified in the title or abstract. When possible, the name of the databases used should be included.	13	(93)	1	(7)	0	(0)	0	(0)
	1.2*		If applicable, the geographical region and timeframe within which the study took place should be reported in the title or abstract.	7	(50)	3	(21)	4	(29)	0	(0)
Introduction											
2		Background/ rationale	Explain the scientific background and rationale for the investigation being reported.	14	(100)	0	(0)	0	(0)	0	(0)
3		Objectives	State specific objectives, including any prespecified hypotheses.	13	(93)	1	(7)	0	(0)	0	(0)
Methods											
4		Study design	Present key elements of study design early in the paper.	11	(79)	3	(21)	0	(0)	0	(0)
5		Setting	Describe the setting, locations, and relevant dates, including periods of recruitment, exposure, follow-up, and data collection.	11	(79)	3	(21)	0	(0)	0	(0)
	7.1*	Variables	A complete list of codes and algorithms used to classify exposures, outcomes, confounders, and effect modifiers should be provided. If these cannot be reported, an explanation should be provided.	1	(7)	2	(14)	11	(79)	0	(0)
	7.1.a**		Describe how the drug exposure definition was developed.	0	(0)	0	(0)	0	(0)	14	(100)
	7.1.b**		Specify the data sources from which drug exposure information for individuals was obtained.	14	(100)	0	(0)	0	(0)	0	(0)
9		Bias	Describe any efforts to address potential sources of bias.	1	(7)	0	(0)	13	(93)	0	(0)
12	(a)	Statistical methods	Describe all statistical methods, including those used to control for confounding.	13	(93)	1	(7)	0	(0)	0	(0)
	(e)		Describe any sensitivity analyses.	1	(7)	0	(0)	0	(0)	13	(93)
	12.1*	Data access	Authors should describe the extent to which the investigators had access to the database.	10	(71)	4	(29)	0	(0)	0	(0)
Results											
13	(c)	Participants	Consider use of a flow diagram.	1	(7)	0	(0)	0	(0)	13	(93)
15		Outcome data	Cohort study—report numbers of outcome events or summary measures over time. Case-control study—report numbers in each exposure category, or summary measures of exposure. Cross sectional study—report numbers of outcome events or summary measures.	13	(93)	1	(7)	0	(0)	0	(0)
17		Other analyses	Report other analyses done—e.g., analyses of subgroups and interactions, and sensitivity analyses.	1	(7)	0	(0)	0	(0)	13	(93)
Discussion											
18		Key results	Summarise key results with reference to study objectives.	4	(29)	3	(21)	4	(29)	3	(21)
19		Limitations	Discuss limitations of the study, taking into account sources of potential bias or imprecision. Discuss both direction and magnitude of any potential bias.	11	(79)	0	(0)	3	(21)	0	(0)
	19.1*		Discuss the implications of using data that were not created or collected to answer the specific research question(s). Include discussion of misclassification bias, unmeasured confounding, missing data, and changing eligibility over time, as they pertain to the study being reported.	8	(57)	3	(21)	3	(21)	0	(0)
	19.1.a**		Describe the degree to which the chosen database(s) adequately captures the drug exposure(s) of interest.	5	(36)	5	(36)	4	(29)	0	(0)
20		Interpretation	Give a cautious overall interpretation of results considering objectives, limitations, multiplicity of analyses, results from similar studies, and other relevant evidence.	10	(71)	4	(29)	0	(0)	0	(0)
21		Generalisability	Discuss the generalisability (external validity) of the study results.	8	(57)	0	(0)	6	(43)	0	(0)
Other information											
22		Funding	Give the source of funding and the role of the funders for the present study and, if applicable, for the original study on which the present article is based.	6	(43)	6	(43)	2	(14)	0	(0)
	22.1*	Accessibility of protocol, raw data, and programming code	Authors should provide information on how to access any supplemental information such as the study protocol, raw data, or programming code.	0	(0)	3	(21)	11	(79)	0	(0)

* Item from the Reporting of studies conducted using observational routinely collected data checklist (RECORD).

** Item from the Reporting of studies conducted using observational routinely collected data for pharmacoepidemiological research checklist (RECORD-PE). Items Nr: 1.3, 4.a, 4.b, 6(a), 6(b), 6.1, 6.2, 6.3, 6.1.a, 7, 7.1.c, 7.1.d, 7.1.e, 7.1.f, 7.1.g, 8, 8.a, 10, 11, 12(b), 12(c), 12(d), 12.1.a, 12.1.b, 12.2, 12.3, 13(a), 13(b), 13.1, 14(a), 14(b), 14(c), 16(a), 16(b), 16(c), 20.a of the checklists were rated by the study authors as being out of scope for the design and type of studies included in this review (e.g. no participants were recruited).

### 3.8. Reported challenges of using web data for drug utilization estimates

Several limitations and biases of using web-mined data for drug utilization estimation were discussed by the study authors. A total of five studies stated that there might be a selection bias as the web data source might not sufficiently represent the whole population and that important vulnerable populations such as the elderly might be underrepresented (21, 23, 29, 31, 33). Furthermore, unmeasured factors, such as users' search intents and attitudes as well as the potential impact of media attention might influence web-mined drug utilization volumes (20, 25, 32). Additional challenges were identified resulting from low search volumes when web data is narrowed down to specific regions or populations (31, 32). In two studies web data was considered to be inadequate to draw causal relationships (20, 25) and it was also stated that web-mined data might generally be unreliable as it is based on self-reported experiences (29).

Four studies specifically addressed limitations of using web-mined data from Google Trends (21, 26, 32, 33). Of these, three studies highlighted that Google Trends only reported a normalized share of the number of searches in the form of “relative search volume” rather than an absolute number of total searches (21, 26, 32). Furthermore, Google Trends provided no details about how research words were recognized or aggregated (33).

## 4. Discussion

This systematic scoping review identified 14 studies which compared drug utilization estimates from web data to another data source. While most studies (13) concluded to some similarities between the two data sources, studies showed a lack of consensus on methodology and only nine (64%) studies used a quantitative measure of correlation between the web and comparison data source.

To our knowledge, this is the only scoping review specifically focusing on the utility of web data for estimating drug utilization in comparison to other data sources. Other recent reviews focused on the use of social media data for pharmacovigilance (8, 34–36), surveillance of prescription medication abuse (37), and illicit drug use (38). Reviews investigating search engine data mostly focused on infectious disease surveillance (39, 40), but, to the best of our knowledge, did not cover the utility for drug utilization so far.

Ultimately, using web data in order to inform on drug utilization could have a significant public health impact. Research is likely to develop in this field showing more examples of association between web data and drug utilization (e.g., types of medication assessed, countries, web data sources used and speed of data obtained) that could confirm our findings.

Our findings are similar to those of a review investigating the utility of social media for pharmacovigilance: Tricco et al. reported consistent results in a majority of included studies which compared the frequency of drug adverse events detected from social media data sources against a regulatory database (8). In addition, our review found that all four included studies that reported on seasonal differences found similar seasonal drug utilization patterns between the two data sources. This finding shows that web data not only generally correlate with comparison data but also underpins the utility of web data to produce timely estimates of drug utilization.

Our review showed a great variety of comparison data sources commonly used for drug utilization studies that were used to validate the results from web data. Those comparison sources included, many country-specific surveillance data sources such as from the US CDC, US Medical Expenditure Panel Surveys (MEPS), and private companies, such as the Japanese JMDC Inc were identified. In these comparison data sources, drug utilization estimates were the most commonly used data measure, before prescription volumes and drug sales.

### 4.1. Web data sources

Twelve (86%) out of 14 included studies employed search engine data retrieved from various Google services such as Google Trends, Google Insights for Search, Google Health Trends, and the Google search engine. Connected to this, the total duration of access was very similar with a median duration of 5.3 years for the web and 5.0 years for the comparison data source. This is notably more than has previously been reported by a review focusing on the utility of social media for pharmacovigilance, where social media posts were followed for a median duration of 1.1 years (8). In addition, the predominance of search engine web data sources might be explained by the greater ease of accessing search engine data through services such as Google Trends compared to retrieving unstructured social media data, which typically involves a labor-intense processing pipeline containing multiple steps (8) to extract datasets suitable for analysis and comparison to other sources. We recommend that research in this field would use a wide range of web data rather than only focussing on one type of research engine (e.g. Facebook, Twitter, specific health forums).

### 4.2. Drug classes and type of drug utilization investigated

Seven out of 14 (50%) studies focused with both antibiotics (*n* = 3) or vaccines (*n* = 4), respectively, on drug classes that belong to the field of infectious diseases. The remaining studies focused on drug classes of diverse other fields, such as diabetes, depression, and the misuse of psychoactive drugs. Studies included medications used either as short treatment (e.g., antibiotics or vaccines) or chronic use (e.g., statins for lipid lower, or antidepressants). However, as most studies used web search engines, they could only evaluate the prevalence of drug use as it is not possible to differentiate former and new users only from these data sources. Using specific analyses of posts content from Facebook, Twitter or specific health forums would allow more information to be retrieved on drug utilization. For instance, one could screen for information on the time patient are on medications or on the concomitant use of other medications. Analysing the content of social media posts has already been used in the past for pharmacovigilance (41). Considering that the investigated studies found consistent positive results of using web data for estimating drug utilization across the vast majority of the investigated drug classes, we advise future studies to extend research to include drug classes from other fields additionally and use a wider diversity of web data sources such as those including specific users posts.

### 4.3. Reported challenges of using web data for drug utilization estimates

The mentioned limitations of the included primary research studies highlighted potential challenges of using web data for estimating drug utilization, such as the potential lack of representativeness between web data-creating users and the general population, difficulties identifying the populations who created the web data, difficulties interpreting relationships between web data and comparison drug utilization data (e.g., due to the presence of potentially unmeasured confounding factors such as users' search intent or effects of media attention), and problems dealing with low search volume if data is narrowed down to specific regions or populations. These critical aspects should be systematically targeted in further studies using web data to assess drug utilization.

### 4.4. Reporting quality

The overall reporting of the studies' quality according to the STROBE, RECORD, and RECORD-PE checklists was mediocre and strongly varied between the different items. The most commonly reported items (>80%) were background/rationale, objectives, and outcome data. Items with low reporting (<20%) were other analyses, bias, and the accessibility of protocol, raw data, and programming code. Of particular relevance is the poor reporting of the two latter items, since both items were rated to be applicable for all reviewed studies and since these points are increasingly recommended as they target research transparency and reproducibility. The finding that articles tend to underreport biases has also been observed in two other studies that assessed the compliance of the articles with the STROBE checklist in different fields (42, 43). One of the issues may be that these guidelines are not specific to internet user content research.

Moreover, many items were rated to be out of scope for the type and design of the studies we included in our review. In many cases, this was due to the fact that the users who created the web data could not directly be regarded as study participants as, for example, eligibility criteria cannot be controlled and important information such as descriptive user characteristics can hardly be retrieved from web data.

In conclusion, the three checklists include all important items necessary to assess the reporting quality of the included studies. However, a variety of items were not applicable as they were out of scope for these types of studies. Therefore, we recommend utilizing a shortened and adapted version of the current STROBE, RECORD, and RECORD-PE checklists for future studies. For example, as web data was usually sourced through social media platforms and open-access websites for search analysis, no actual participant recruitment procedures took place in those studies. Therefore, all items relating to the recruitment and assessment of real-world participants could be omitted in a future version of this checklist (i.e., items: 6(a), 6(b), 6.1, 6.2, 6.3, 6.1.a, 13(a), 13(b), 13.1, 14(a), 14(b), 14(c)) and replaced by more suited item such as: the type of web data (e.g. search terms volumes, number of tweets/posts of interest…).

### 4.5. Strengths and limitations

This systematic scoping review was conducted and reported according to the standardized PRISMA guidelines (15). We conducted an extensive literature search, defined the study eligibility criteria, rigorously assessed studies that contained drug utilization information from web data sources, and compared it to other sources with drug utilization information.

One limitation of this review was the heterogeneity of methodologies in terms of study objectives and analysis methods in the included studies, which made it impossible to draw more general conclusions. This, together with the relatively small number of identified studies, underlines the complexity and novelty of the field and justifies the selection of a scoping review approach.

Finally, in our assessment of the studies' reporting quality employing the STROBE, RECORD, and RECORD-PE checklist, a substantial number of items had to be considered out of scope for these types of studies. This requests for an adapted (standard) checklist.

## 5. Conclusion

While this study demonstrates the potential of social media and search engine data in assessing drug utilization, it also emphasizes the low level of evidence available in the literature. Generalization of this approach requires additional studies focusing on the validation of drug utilization estimates from traditional data sources as well as on using quantitative (such as correlation assessment or modelling) methodologies when comparing traditional sources to web data. The use of web data to estimate drug utilization is an emerging field, and future research should focus on fulfilling standardized reporting standards as well as developing new reporting guidelines that specifically target the characteristics of this type of research.

## Supplementary material

**File 1**

Research protocol

**File 2**

Sample search strategy

**File 3**

Excluded studies with reasons

**File 4**

Data extraction of included studies

**File 5**

Filled in PRISMA ScR checklist
