# Supplementary material for: Social media and internet search data to inform drug utilization: A systematic scoping review
Source: Front Digit Health. 2023 Mar 20;5:1074961. doi: 10.3389/fdgth.2023.1074961 (PMC10067924; doi:10.3389/fdgth.2023.1074961)
Supplement: Supplementary file 1 [file Datasheet1.docx]

**STUDY PROTOCOL**

**Keller et al., 2023**

Adopted from PRISMA-ScR (Preferred Reporting Items for Systematic review and Meta-Analyses extension for Scoping Reviews Protocols) and PROSPERO

| Topic | Content |
| --- | --- |
| Title | Social Media and Internet Search Data may be used to follow Drug use in Real-Time: A Systematic Scoping Review of Evidence prior to the Covid19 Pandemic |
| Authors | Roman Keller, Alessandra Spanu, Milo Alan Puhan, Antoine Flahault, Christian Lovis, Margot Mütsch*, Raphaelle Beau-Lejdstrom*  *One shared last authorship |
| Review team members and their organizational affiliations | Roman Keller, Epidemiology, Biostatistics and Prevention Institute, University of Zurich, Zurich, Switzerland Alessandra Spanu, Epidemiology, Biostatistics and Prevention Institute, University of Zurich, Zurich, Switzerland Milo Alan Puhan, Epidemiology, Biostatistics and Prevention Institute, University of Zurich, Zurich, Switzerland Antoine Flahault, Institute of Global Health, University of Geneva, Geneva, Switzerland Christian Lovis, Department of Radiology and Medical Informatics, University of Geneva, Geneva, Switzerland Margot Mütsch, Epidemiology, Biostatistics and Prevention Institute, University of Zurich, Zurich, Switzerland  Raphelle Beau-Lejdstrom, Epidemiology, Biostatistics and Prevention Institute, University of Zurich, Zurich, Switzerland |
| Contact details for of corresponding author | Roman Keller Future Health Technologies Programme Campus for Research Excellence and Technological Enterprise Singapore-ETH Centre 1 Create Way CREATE Tower, #06-01 Singapore Email: roman.keller@sec.ethz.ch |
| Organizational affiliation of the review | Epidemiology, Biostatistics and Prevention Institute, University of Zurich, Zurich, Switzerland  Singapore-ETH Centre, Future Health Technologies Programme, CREATE Campus, Singapore, Singapore  Saw Swee Hock School of Public Health, National University of Singapore, Singapore  Institute of Global Health, University of Geneva, Geneva, Switzerland Department of Radiology and Medical Informatics, University of Geneva, Geneva, Switzerland |
| Type and method of review | Systematic scoping review |
| Contributions | Study design: AS, RB, MM, CL, AF, MP; Search strategy: AS, RB, MM; Screening: RK, AS, RB; Data extraction: RK, AS; Data analysis: RK; First draft: RK; Revisions and subsequent drafts: RK, AS, MM, RB; Critical feedback for final draft: RB, MM, MP |
| Sources/Sponsors | RK received funding by the University of Zurich; AS received PhD funding by the University of Zurich. 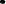 |
| Conflict of interest | None |
| Rationale | Web data comprising social media and internet search data such as from Facebook, Twitter or Google Trends are increasingly used in order to identify geospatial distribution of epidemics and other diseases. More recently, research has been mainly focused on detecting adverse events signals from social media. The association between drug use in web data and other more classical drug utilization data sources has not been clearly demonstrated.  Information on drug utilization is usually difficult to access through traditional research methods (drug sales, prescription volumes or Electronic Health Records EHR) and the use of web data could provide an easier and more timely access information on medications’ utilization worldwide.  We aim to assess quality and content of existing research using Facebook, Twitter, Instagram or Google Trends/Google Insights for Search /Google AdWords to study drug utilization volumes and compare them to other sources of drug utilization. We will explore if current research offers sufficient data for exploiting these novel databases, challenge the quality of findings and identify main gaps to fill in this area. |
| Eligibility criteria | Inclusion criteria:   1. Primary research studies that involved a data source of web data including social media or search engine data such as Google Trends, Google Correlate, Google Insights for Search, Google search engine, Facebook, Twitter or Instagram 2. Involvement of another validated data source such as drug sales or prescription volumes acquired from surveys, registry data or physician databases 3. Inclusion of any kind of drug utilization data such as utilization frequencies of vaccines, vitamins, supplements, nicotine alternatives, prescription drugs or over-the-counter drugs for both data sources   Exclusion criteria:   1. Focus on E-cigarettes 2. Studies involving incidence rates of diseases instead of than medication volumes 3. Involvement of two data sources that are both categorized as unvalidated web data sources by the study authors and where no validated data source is available 4. Conference papers, workshop papers, literature reviews, posters, power point presentations, articles presented at doctoral colloquia, or if the article’s full text was not accessible for the study authors 5. Non-English study documents |
| Information sources | A database search will be conducted accessing PubMed Medline, EMBASE, Scopus, and Web of Science. These databases were chosen as the cover relevant topics in health sciences (PubMed Medline, EMBASE) and interdisciplinary research (WOS and Scopus). Search terms include synonyms, acronyms, and commonly known terms of the constructs “Google Trends”, “Google Correlate”, “Google Insights”, “Google search engine”, “Facebook”, “Twitter”, “Instagram”, “drug sales”, “prescription volume”, “vaccines”, “vitamins”, “supplements”, “nicotine alternatives”, “prescription drugs” and “over-the-counter drugs”. Grey literature such as posters, presentations, dissertations, and theses will be excluded. |
| Search strategy | Search strategy for PubMed Medline  (https://pubmed.ncbi.nlm.nih.gov/)  Filters: none  Conducted in September 2016   \| 1. exp drug therapy/ \|  \| \| --- \| --- \|  \| 2. "drug*".ab,kw,ti. \|  \| \| --- \| --- \|  \| 3. "treatment*".ab,kw,ti. \|  \| \| --- \| --- \|  \| 4. "medication*".ab,kw,ti. \|  \| \| --- \| --- \|  \| 5. exp vaccines/ \|  \| \| --- \| --- \|  \| 6. exp vitamins/ \|  \| \| --- \| --- \|  \| 7. exp herbal medicine/ \|  \| \| --- \| --- \|  \| 8. exp plant extracts/ \|  \| \| --- \| --- \|  \| 9. exp phytotherapy/ \|  \| \| --- \| --- \|  \| 10. exp "Tobacco Use Cessation Products"/ \|  \| \| --- \| --- \|  \| 11. google insight$.tw. \|  \| \| --- \| --- \|  \| 12. google trend$.tw. \|  \| \| --- \| --- \|  \| 13. twitter.tw. \|  \| \| --- \| --- \|  \| 14. facebook.tw. \|  \| \| --- \| --- \|  \| 15. (tweet* not "tweetable abstract").tw. \|  \| \| --- \| --- \|  \| 16. exp drug utilization/ \|  \| \| --- \| --- \|  \| 17. exp immunization/ \|  \| \| --- \| --- \|  \| 18. surveillance.ab,kw,ti. \|  \| \| --- \| --- \|  \| 19. exp drug information services/ \|  \| \| --- \| --- \|  \| 20. (google adj3 trend$).tw. \|  \| \| --- \| --- \|  \| 21. (google adj3 insight$).tw. \|  \| \| --- \| --- \|  \| 22. vaccin*.ab,kw,ti. \|  \| \| --- \| --- \|  \| 23. vitamin*.ab,kw,ti. \|  \| \| --- \| --- \|  \| 24. "herbal medicine".ab,kw,ti. \|  \| \| --- \| --- \|  \| 25. "Tobacco Use Cessation Products".ab,kw,ti. \|  \| \| --- \| --- \|  \| 26. ("e-cigarette*" or "e cigarette*" or "Electronic Nicotine Delivery System*").ab,kw,ti. \|  \| \| --- \| --- \|  \| 27. "drug utili*ation".ab,kw,ti. \|  \| \| --- \| --- \|  \| 28. "immuni*ation".ab,kw,ti. \|  \| \| --- \| --- \|  \| 29. exp product surveillance, post marketing/ \|  \| \| --- \| --- \|  \| 30. exp "Drug-Related Side Effects and Adverse Reactions"/ \|  \| \| --- \| --- \|  \| 31. "adverse effect*".ab,ti,kw. \|  \| \| --- \| --- \|  \| 32. exp pharmaceutical preparations/ \|  \| \| --- \| --- \|  \| 33. 11 or 12 or 13 or 14 or 15 or 20 or 21 \|  \| \| --- \| --- \|  \| 34. phytotherap*.ab,kw,ti. \|  \| \| --- \| --- \|  \| 35. "plant extract*".ab,kw,ti. \|  \| \| --- \| --- \|  \| 36. monitoring.ab,kw,ti. \|  \| \| --- \| --- \|  \| 37. "pharmaceutical preparations".ti,ab,kw. \|  \| \| --- \| --- \|  \| 38. 1 or 2 or 3 or 4 or 5 or 6 or 7 or 8 or 9 or 10 or 16 or 17 or 18 or 19 or 22 or 23 or 24 or 25 or 26 or 27 or 28 or 29 or 30 or 31 or 32 or 34 or 35 or 36 or 37 \|  \| \| --- \| --- \|  \| 39. 33 and 38 \|  \| \| --- \| --- \|  \| 40. instagram$.tw. \|  \| \| --- \| --- \|  \| 41. 38 or 40 \|  \| \| --- \| --- \|  \| 42. 33 and 41 \| \| --- \| |
| Type of included study | Any type of primary research |
| Studied domain | Pharmacoepidemiology |
| Population/Participants | Any population and any participants (clinical / non-clinical) |
| Data collection and selection process | Two independent reviewers will conduct the initial screening of the obtained studies based on their titles and abstracts. The same reviewers will then independently conduct full-text screening based on the eligibility/inclusion criteria including a Cohen’s kappa computation (assessed for title and abstract screening, full text screening) with the search update to measure interrater reliability. Any disagreement will be discussed in person. If no consensus can be reached, the discussion will be brought to another investigator to achieve agreement. |
| Data items for coding | The following data items will be extracted of each included study: first author, year of publication, country of first author, study objective, study design, web data source, comparison data source, measure in web data source, measure in comparison data source, statistics of comparison, population of interest, time period assessed, total duration assessed, medication of interest, main findings, locations of data origin, funding sources, conflicts of interest. Further items concerning the “STrengthening the Reporting of OBservational studies in Epidemiology” (STROBE), the “REporting of studies Conducted using Observational Routinely-collected Data” (RECORD) as well as the “REporting of studies Conducted using Observational Routinely-collected Data for pharmacoepidemiology” (RECORD-PE) checklist will be extracted according to the checklist items’ applicability to the included documents.  One independent reviewer will conduct the data extraction which will be spot-checked by a second independent reviewer. Any uncertainties will be discussed and resolved in consensus. |
| Outcomes and prioritization | Main outcomes: Frequencies of drug utilization. Frequencies can be retrieved through web data sources (e.g. drug search volumes, number of posts on drug consumption, drug query indexes) or validated data sources (e.g. drug prescription volumes, official drug utilization estimates, drug sales). |
| Risk of bias in individual studies | Risk of bias assessment will not be conducted which is consistent with the Joanna Briggs Institute methods manual (methodology for JBI Scoping reviews). |
| Data synthesis | The PRISMA-ScR statement will be followed for data synthesis. A narrative synthesis of the included studies will be performed. |
| Language | English |
| Country | Switzerland |
| Anticipated or actual start date | September 2016 |
| Anticipated or actual end date | August 2022 |

Adopted from:

Tricco AC, Lillie E, Zarin W, et al. PRISMA Extension for Scoping Reviews (PRISMA-ScR): Checklist and Explanation. *Ann Intern Med*. 2018;169(7):467.

Booth A, Clarke M, Dooley G, et al. The nuts and bolts of PROSPERO: An international prospective register of systematic reviews [Internet]. *Syst Rev*. 2012;1(1) doi:10.1186/2046-4053-1-2.

Peters MDJ, Godfrey CM, Khalil H, McInerney P, Parker D, Soares CB. Guidance for conducting systematic scoping reviews. *Int J Evid Based Healthc*. 2015;13(3):141–6.
