# Supplementary material for: Social media and internet search data to inform drug utilization: A systematic scoping review
Source: Front Digit Health. 2023 Mar 20;5:1074961. doi: 10.3389/fdgth.2023.1074961 (PMC10067924; doi:10.3389/fdgth.2023.1074961)
Supplement: Supplementary file 2 [file Datasheet2.docx]

# Supplementary Box: MEDLINE search strategy

| Search strategy for PubMed Medline  (https://pubmed.ncbi.nlm.nih.gov/)  Filters: none  Searched: Sept 6^th^ 2016  Records retrieved: 536   \| 1. exp drug therapy/ \|  \| \| --- \| --- \|  \| 2. "drug*".ab,kw,ti. \|  \| \| --- \| --- \|  \| 3. "treatment*".ab,kw,ti. \|  \| \| --- \| --- \|  \| 4. "medication*".ab,kw,ti. \|  \| \| --- \| --- \|  \| 5. exp vaccines/ \|  \| \| --- \| --- \|  \| 6. exp vitamins/ \|  \| \| --- \| --- \|  \| 7. exp herbal medicine/ \|  \| \| --- \| --- \|  \| 8. exp plant extracts/ \|  \| \| --- \| --- \|  \| 9. exp phytotherapy/ \|  \| \| --- \| --- \|  \| 10. exp "Tobacco Use Cessation Products"/ \|  \| \| --- \| --- \|  \| 11. google insight$.tw. \|  \| \| --- \| --- \|  \| 12. google trend$.tw. \|  \| \| --- \| --- \|  \| 13. twitter.tw. \|  \| \| --- \| --- \|  \| 14. facebook.tw. \|  \| \| --- \| --- \|  \| 15. (tweet* not "tweetable abstract").tw. \|  \| \| --- \| --- \|  \| 16. exp drug utilization/ \|  \| \| --- \| --- \|  \| 17. exp immunization/ \|  \| \| --- \| --- \|  \| 18. surveillance.ab,kw,ti. \|  \| \| --- \| --- \|  \| 19. exp drug information services/ \|  \| \| --- \| --- \|  \| 20. (google adj3 trend$).tw. \|  \| \| --- \| --- \|  \| 21. (google adj3 insight$).tw. \|  \| \| --- \| --- \|  \| 22. vaccin*.ab,kw,ti. \|  \| \| --- \| --- \|  \| 23. vitamin*.ab,kw,ti. \|  \| \| --- \| --- \|  \| 24. "herbal medicine".ab,kw,ti. \|  \| \| --- \| --- \|  \| 25. "Tobacco Use Cessation Products".ab,kw,ti. \|  \| \| --- \| --- \|  \| 26. ("e-cigarette*" or "e cigarette*" or "Electronic Nicotine Delivery System*").ab,kw,ti. \|  \| \| --- \| --- \|  \| 27. "drug utili*ation".ab,kw,ti. \|  \| \| --- \| --- \|  \| 28. "immuni*ation".ab,kw,ti. \|  \| \| --- \| --- \|  \| 29. exp product surveillance, post marketing/ \|  \| \| --- \| --- \|  \| 30. exp "Drug-Related Side Effects and Adverse Reactions"/ \|  \| \| --- \| --- \|  \| 31. "adverse effect*".ab,ti,kw. \|  \| \| --- \| --- \|  \| 32. exp pharmaceutical preparations/ \|  \| \| --- \| --- \|  \| 33. 11 or 12 or 13 or 14 or 15 or 20 or 21 \|  \| \| --- \| --- \|  \| 34. phytotherap*.ab,kw,ti. \|  \| \| --- \| --- \|  \| 35. "plant extract*".ab,kw,ti. \|  \| \| --- \| --- \|  \| 36. monitoring.ab,kw,ti. \|  \| \| --- \| --- \|  \| 37. "pharmaceutical preparations".ti,ab,kw. \|  \| \| --- \| --- \|  \| 38. 1 or 2 or 3 or 4 or 5 or 6 or 7 or 8 or 9 or 10 or 16 or 17 or 18 or 19 or 22 or 23 or 24 or 25 or 26 or 27 or 28 or 29 or 30 or 31 or 32 or 34 or 35 or 36 or 37 \|  \| \| --- \| --- \|  \| 39. 33 and 38 \|  \| \| --- \| --- \|  \| 40. instagram$.tw. \|  \| \| --- \| --- \|  \| 41. 38 or 40 \|  \| \| --- \| --- \|  \| 42. 33 and 41 \| \| --- \| |
| --- | --- | --- | --- | --- | --- | --- | --- | --- | --- | --- | --- | --- | --- | --- | --- | --- | --- | --- | --- | --- | --- | --- | --- | --- | --- | --- | --- | --- | --- | --- | --- | --- | --- | --- | --- | --- | --- | --- | --- | --- | --- | --- | --- | --- | --- | --- | --- | --- | --- | --- | --- | --- | --- | --- | --- | --- | --- | --- | --- | --- | --- | --- | --- | --- | --- | --- | --- | --- | --- | --- | --- | --- | --- | --- | --- | --- | --- | --- | --- | --- | --- | --- | --- |
