# Supplementary material for: Social media and internet search data to inform drug utilization: A systematic scoping review
Source: Front Digit Health. 2023 Mar 20;5:1074961. doi: 10.3389/fdgth.2023.1074961 (PMC10067924; doi:10.3389/fdgth.2023.1074961)
Supplement: Supplementary file 3 [file Datasheet3.docx]

# Supplementary Table: Excluded Studies

The most common reason for exclusion was wrong study design (70 papers) such as the lack of a comparison data source. Other common reasons were; irrelevant or wrong outcomes (22 papers), i.e. not data on drug utilization, wrong setting (6 papers), such as papers that used questionnaires on social media, others (2 paper), and studies without available full-text (23 papers), such as conference abstracts.

|  | **AUTHORS** | **TITLE** | **JOURNAL** | **CRITERIA** |
| --- | --- | --- | --- | --- |
| 1 | G. B. Powell, H; Rodriguez, H; Thomas, M; Painter, J; Harvey, C; Blowers, J; Dasgupta, N | Comparison of events in spontaneous adverse event reports to events discussed within context of drug use on facebook and twitter | Pharmacoepidemiology and Drug Safety | No full-text available |
| 2 | N. P. Dasgupta, C; Xu, E; Menone, C M; Freifeld, C F; Rodriguez, H; Heffernan, R; Bahk, C; Brownstein, J S | Does direct-to-consumer advertising lead to stimulated reporting of adverse events in social media? | Pharmacoepidemiology and Drug Safety | No full-text available |
| 3 | M. S. C. Duh, P.; Van Audenrode, M.; Vekeman, F.; Karner, P.; Giguere-Duval, P.; Xiao, Y.; Damron, J.; Greenberg, P. | Evaluation of the value of web data for detecting drug adverse events | Pharmacoepidemiology and Drug Safety | No full-text available |
| 4 | V. L.-L. L. Koutkias, A; Jaulent, M C | Exploiting heterogeneous open data for pharmacovigilance | Drug Safety | No full-text available |
| 5 | V. M. König, R. | Google® knows when you will sneezing | Allergy: European Journal of Allergy and Clinical Immunology | No full-text available |
| 6 | J. C. P.-G. Juárez-Giménez, C.; Pérez-Ricart, A.; Lalueza-Broto, P.; Girona-Brumos, L. | Professional use of twitter for drug information | European Journal of Hospital Pharmacy | No full-text available |
| 7 | M. D. M. Toussi, C.; Nadarajah, S.; Breckenridge, A. | Tracking of adverse drug reactions in social media: Current status of requirements, best practices, methods and tools | Pharmacoepidemiology and Drug Safety | No full-text available |
| 8 | P. M. B. Coloma, B.; Van Mulligen, E.; Kors, J. A. | Use of social media in pharmacovigilance: Testing the waters | Pharmacoepidemiology and Drug Safety | No full-text available |
| 9 | A. Kitta | Using web 2.0 technologies to understand public concerns and provide information | Canadian Journal of Infectious Diseases and Medical Microbiology | No full-text available |
| 10 | B. D. Dreyfus, J.; Gomez-Caminero, A. | Utility of social media surveillance for drug safety and signal detection purposes | Pharmacoepidemiology and Drug Safety | No full-text available |
| 11 | P. M. B. Coloma, B.; Sturkenboom, M. C. J. M.; Van Mulligen, E. M.; Kors, J. A. | What can social media networks contribute to medicines safety surveillance? | Pharmacoepidemiology and Drug Safety | No full-text available |
| 12 | G. R.-V. Gimenez-Perez, M.; Recasens, A.; Simó, O.; Castells, I. | Who is using Twitter to communicate about diabetes and what for? | Diabetologia | No full-text available |
| 13 | Dasgupta, N; Pierce, C E; Bouri, K; Pamer, C; Proestel, S; Rodriguez, H W; Van Le, H; Freifeld, C C; Brownstein, J S; Walderhaug, M; Edwards, I R | Can facebook and twitter monitoring yield earlier detection of safety signals for medical products? | | No full-text available |
| 14 | Dreyfus, B; Pierce, C E | Social media compared to faers and administrative claims for pharmacovigilance | | No full-text available |
| 15 | Harpster, E; Hultgren, K | Ciprofloxacin and levofloxacin: Twitter versus food and drug administration adverse event reporting system | | No full-text available |
| 16 | Hussain, S; Belkind-Gerson, J; Chogle, A; Hicks, T; Bhuiyan, M; Misra, S | The peg controversy:-caregivers, doctors and the internet |  | No full-text available |
| 17 | Korenoski, A; Smith, R; Lynch, M | Do internet search query trends relate to intentional gabapentin exposures reported to U.S. poison centers? | | No full-text available |
| 18 | Saulsberry, L; Viswanath, V | A TWITTER ANALYSIS OF MEASLES AND VACCINATION CONVERSATION DURING THE 2015 MEASLES OUTBREAK | | No full-text available |
| 19 | Ortiz, R; Coyne-Beasley, T; Cates, J; Shafer, A | ENTERTAIN THEM WHERE THEY ARE: TESTING THE FEASIBILITY AND EFFECTIVENESS OF A FACEBOOK INTERVENTION TO INCREASE HPV VACCINE KNOWLEDGE AND VACCINATION INTENTIONS AMONG ADOLESCENTS | | No full-text available |
| 20 | Patel, S K; Amos, A; Desai, S; Amos, D | Understanding adherence to AAD treatment guidelines for acne vulgaris via analysis of Google search trends | | No full-text available |
| 21 | Smith, K L; Sarker, A; Nikfarjam, A; Malone, D; Gonzalez-Hernandez, G | Mining adverse events in twitter: Experiences of adalimumab users |  | No full-text available |
| 22 | Walker, A L; Farzan, R; Gaydos, L M; De Castro, L; Jonassaint, C | Assessing perceptions of hydroxyurea among sickle cell disease stakeholders using social media | | No full-text available |
| 23 | Turakhia, M.P.; Harrington, R.A. | Twitter and cardiovascular disease: Useful chirps or noisy chatter? |  | No full-text available |
| 24 | X. Huang, M.C. Smith, M.J. Paul, D. Ryzhkov, S.C. Quinn, D.A. Broniatowski, M.Dredze | Examining Patterns of Influenza Vaccination in Social Media | The AAAI-17 Joint Workshop on Health Intelligence WS-17-09 | Other |
| 25 | not set | Most tweets are positive |  | Other |
| 26 | MacKinlay, A.; Aamer, H.; Yepes, A.J. | Detection of Adverse Drug Reactions using Medical Named Entities on Twitter | | Wrong outcomes |
| 27 | Smith, K.; Golder, S.; Sarker, A.; Loke, Y.; O’Connor, K.; Gonzalez-Hernandez, G. | Methods to Compare Adverse Events in Twitter to FAERS, Drug Information Databases, and Systematic Reviews: Proof of Concept with Adalimumab | | Wrong outcomes |
| 28 | Bonaldo, G; Vaccheri, A; D'Annibali, O; Motola, D | Safety profile of human papilloma virus vaccines: an analysis of the US Vaccine Adverse Event Reporting System from 2007 to 2017 | | Wrong outcomes |
| 29 | Kim, S J; Schiffelbein, J E; Imset, I; Olson, A L | FOCUS GROUPS ON HPV VACCINE VIA FACEBOOK ADS: IDENTIFYING THEMES AND LINGUISTIC CHARACTERISTICS OF USER COMMENTS | | Wrong outcomes |
| 30 | Niforatos, J D; Zheutlin, A R; Pescatore, R M; Raja, A S | Public interest in medication-assisted treatment for opioid used disorder in the United States | | Wrong outcomes |
| 31 | Patel, R; Belousov, M; Jani, M; Dasgupta, N; Winokur, C; Nenadic, G; Dixon, W G | Frequent discussion of insomnia and weight gain with glucocorticoid therapy: An analysis of Twitter posts | | Wrong outcomes |
| 32 | Maskell, S | When does social media add value to pharmacovigilance? |  | Wrong outcomes |
| 33 | Mitchell, L; Ross, J V | A data-driven model for influenza transmission incorporating media effects | | Wrong outcomes |
| 34 | Tanti, M; Fossey, S; Madrid-Briand, L; Carrieri, P; Spire, B; Roux, P | An Analysis of Twitter for Better Understanding of Communication Actors in New Drugs and their Discussion | | Wrong outcomes |
| 35 | Yadav, S; Ekbal, A; Saha, S; Bhattacharyya, P; Acl | A Unified Multi-task Adversarial Learning Framework for Pharmacovigilance Mining | | Wrong outcomes |
| 36 | Young, L E; Fujimoto, K; Schneider, J A | Facebook group affiliation ties, group topics, and HIV behavioral characteristics among young Black men who have sex with men: Potential for public health intervention | | Wrong outcomes |
| 37 | Gupta, S; Gupta, M; Varma, V; Pawar, S; Ramrakhiyani, N; Palshikar, G K | No Title |  | Wrong outcomes |
| 38 | W. P. Jackson, B.; Toscani, M.; Hermes-DeSantis, E. | Analysis of Social Media Interactions Between Pharmaceutical Companies and Consumers: The Power of the “Like” | Therapeutic Innovation and Regulatory Science | Wrong outcomes |
| 39 | C. C. B. Freifeld, J S; Menone, C M; Bao, W; Filice, R; Kass-Hout, T; Dasgupta, N | Digital drug safety surveillance: Monitoring pharmaceutical products in Twitter | Drug Safety | Wrong outcomes |
| 40 | A. T. Volovyk, O. | Feasibility of conducting retrospective studies using hashtags and social media data from facebook and twitter | Value in Health | Wrong outcomes |
| 41 | L. Rossignol, C. Pelat, B. Lambert, A. Flahault, E. Chartier-Kastler, T. Hanslik | A Method to Assess Seasonality of Urinary Tract Infections Based on Medication Sales and Google Trends | PLoS ONE | Wrong outcomes |
| 42 | A. Q. Zheluk, C.; Meylakhs, P. | Internet search and krokodil in the Russian Federation: an infoveillance study | Journal of Medical Internet Research | Wrong outcomes |
| 43 | L. P. Orsolini, G. D.; Francesconi, G.; Schifano, F. | Mind navigators of chemicals' experimenters? A web-based description of e-psychonauts | Cyberpsychology, behavior and social networking | Wrong outcomes |
| 44 | E. M. Schaeffer | Re: A method to assess seasonality of urinary tract infections based on medication sales and google trends: Editorial comment | Journal of Urology | Wrong outcomes |
| 45 | Garrison, S. R. Dormuth, C. R. Morrow, R. L. Carney, G. A. Khan, K. M. | Seasonal effects on the occurrence of nocturnal leg cramps: a prospective cohort study | Cmaj | Wrong outcomes |
| 46 | B. F. Stone, J.; Lee, R.; Te, A.; Chughtai, B. | The impact on internet search activity and media coverage after the FDA safety communication on surgical mesh for pelvic organ prolapse | Neurourology and Urodynamics | Wrong outcomes |
| 47 | N. P. Dasgupta, C | Use of social media for data mining in pharmacovigilance | Drug Safety | Wrong outcomes |
| 48 | Q. L. Boucherie, L; Bonin-Guillaume, S; Blin, O; Micallef-Roll, J | Trends in use of meprobamate in older people: Impact of Health Authorities measures | Fundamental and Clinical Pharmacology | Wrong setting |
| 49 | Ravoire, S; Lang, M; Perrin, E; Audry, A; Bilbault, P; Chekroun, M; Demerville, L; Escudier, T; Gueroult-Accolas, L; Guillot, C; Malbezin, M; Maugendre, P; Micallef, J; Molimard, M; Montastruc, F; Pierron, E; Reichardt, L; Thiessard, F | Interests and limitations of virtual patient communities for the research in health products | | Wrong setting |
| 50 | D. S. Scanfeld, V; Larson, E L | Dissemination of health information through social networks: Twitter and antibiotics | American Journal of Infection Control | Wrong setting |
| 51 | V. G. Risson, B.; Bonzani, I.; Korn, J. R.; Medin, J.; Olson, M. S. | Linkage analysis of multiple-sclerosis patient data from social-media surveys and claims databases identifies a representative population for real-life outcomes research | Value in Health | Wrong setting |
| 52 | V. S. Risson, D.; Bonzani, I.; Huisman, A.; Olson, M. | Patterns of Treatment Switching in Multiple Sclerosis Therapies in US Patients Active on Social Media: Application of Social Media Content Analysis to Health Outcomes Research | Journal of Medical Internet Research | Wrong setting |
| 53 | O. A. Corazza, S.; Simonato, P.; Corkery, J.; Bersani, F. S.; Demetrovics, Z.; Stair, J.; Fergus, S.; Pezzolesi, C.; Pasinetti, M.; Deluca, P.; Drummond, C.; Davey, Z.; Blaszko, U.; Moskalewicz, J.; Mervo, B.; Furia, L. D.; Farre, M.; Flesland, L.; Pisarska, A.; Shapiro, H.; Siemann, H.; Skutle, A.; Sferrazza, E.; Torrens, M.; Sambola, F.; van der Kreeft, P.; Scherbaum, N.; Schifano, F. | Promoting innovation and excellence to face the rapid diffusion of novel psychoactive substances in the EU: the outcomes of the ReDNet project | Human Psychopharmacology | Wrong setting |
| 54 | Jouanjus, E.; Mallaret, M.; Micallef, J.; Ponté, C.; Roussin, A.; Lapeyre-Mestre, M. | Comment on: "Social Media Mining for Toxicovigilance: Automatic Monitoring of Prescription Medication Abuse from Twitter" | | Wrong study design |
| 55 | Shang, W.; Chen, H.; Livoti, C. | Adverse drug reaction early warning using user search data |  | Wrong study design |
| 56 | Nguyen, T.; Larsen, M.E.; O'Dea, B.; Phung, D.; Venkatesh, S.; Christensen, H. | Estimation of the prevalence of adverse drug reactions from social media | | Wrong study design |
| 57 | Ortiz-Martínez, Y.; Galindo-Regino, C.; Valdes-Villegas, F.; Mendoza-Borja, K.; González-Hurtado, M.R.; Chávez-Verbel, V. | World Antibiotic Awareness Week 2017 and its influence on digital information seeking on antibiotic resistance: A Google Trends study | | Wrong study design |
| 58 | Suragh, T.A.; Lamprianou, S.; MacDonald, N.E.; Loharikar, A.R.; Balakrishnan, M.R.; Benes, O.; Hyde, T.B.; McNeil, M.M. | Cluster anxiety-related adverse events following immunization (AEFI): An assessment of reports detected in social media and those identified using an online search engine | | Wrong study design |
| 59 | Menzies, S.; Daly, S.; McKenna, D.B. | Social media and psoriasis treatment: what are people saying on Twitter? | | Wrong study design |
| 60 | Santillana, M. | Perspectives on the future of internet search engines and biosurveillance systems | | Wrong study design |
| 61 | Peng, Y.; Moh, M.; Moh, T.-S. | Efficient adverse drug event extraction using Twitter sentiment analysis | | Wrong study design |
| 62 | Sarker, A.; Malone, D.; Gonzalez, G. | Authors’ Reply to Jouanjus and Colleagues’ Comment on “Social Media Mining for Toxicovigilance: Automatic Monitoring of Prescription Medication Abuse from Twitter” | | Wrong study design |
| 63 | Brijbassi, M.; Anand, A.; Stanbrook, M. | The TRINITY study: Twitter discussion from a respirology journal club |  | Wrong study design |
| 64 | Bloomgarden, Z | Talks and tweets from the ADA |  | Wrong study design |
| 65 | Duval, F V; Silva, Fabd | Mining in Twitter for adverse events from malaria drugs: the case of doxycycline | | Wrong study design |
| 66 | Miller, J | Innovation vs. Capacity: How CMOs compete |  | Wrong study design |
| 67 | Miller, J | CDMO acquisitions build strategic supplier base |  | Wrong study design |
| 68 | Miller, J | CMOS and CROs have different trajectories |  | Wrong study design |
| 69 | Miller, J | Will pharma manufacturing move back to the United States? |  | Wrong study design |
| 70 | Miller, J | The tide stays high |  | Wrong study design |
| 71 | Platkin, C; Link, A R; Kwan, A | The Digital Revolution and Its Potential Impact on Detection and Treatment of Depressive Disorders | | Wrong study design |
| 72 | Rosenbaum, L | Twitter tailwinds - Little capsules of gratitude |  | Wrong study design |
| 73 | Shah, M; Lopman, B; Tate, J; Harris, J; Esparza-Aguilar, M; Sanchez-Uribe, E; Richardson, V; Parashar, U D | Use of internet search data to monitor rotavirus vaccine impact in The United States, United Kingdom and Mexico | | Wrong study design |
| 74 | Shankar, P R | Commentary on Chemotherapy |  | Wrong study design |
| 75 | Thomas, R B; Johnson, P T; Fishman, E K | Social Media for Global Education: Pearls and Pitfalls of Using Facebook, Twitter, and Instagram | | Wrong study design |
| 76 | Tibebu, S; Chang, V C; Drouin, C A; Thompson, W; Do, M T | Aperçu - Que révèlent les médias sociaux au sujet de la crise des opioïdes au Canada? | | wrong study design |
| 77 | Timothy, P G; Jeffrey, B; Kaitlyn, L; Margarita, V D | Delivery of educational content via Instagram(®) |  | Wrong study design |
| 78 | Y. L. Aphinyanaphongs, S.; Nguyen, V.; Nelson, L.; Krebs, P.; Su, M.; Smith, S. W. | A pilot application of automatic tweet detection of alcohol use at a music festival | Annals of Emergency Medicine | Wrong study design |
| 79 | J. Z. Liu, S.; Zhang, X. | An ensemble method for extracting adverse drug events from social media | Artificial Intelligence in Medicine | Wrong study design |
| 80 | C. L. C. Hanson, B.; Burton, S.; Giraud-Carrier, C. | An exploration of social circles and prescription drug abuse through Twitter | Journal of Medical Internet Research | Wrong study design |
| 81 | C. B. R. Whitman, M. W.; Arnold, C.; Patel, H.; Ursos, L.; Sa'adon, R.; Pourmorady, J.; Spiegel, B. M. | Balancing opioid-induced gastrointestinal side effects with pain management: Insights from the online community | Journal of Opioid Management | Wrong study design |
| 82 | F. S. Gesualdo, G.; D'Ambrosio, A.; Carloni, E.; Pandolfi, E.; Velardi, P.; Fiocchi, A.; Tozzi, A. E. | Can Twitter Be a Source of Information on Allergy? Correlation of Pollen Counts with Tweets Reporting Symptoms of Allergic Rhinoconjunctivitis and Names of Antihistamine Drugs | PLoS ONE [Electronic Resource] | Wrong study design |
| 83 | S. J. J. Yea, Y.; Seong, B.; Kim, C. | Comparative analysis of web search trends between experts and public for medicinal herbs in Korea | J Ethnopharmacol | Wrong study design |
| 84 | N. C. Alvaro, M.; Doan, S.; Lofi, C.; Overington, J.; Collier, N. | Crowdsourcing Twitter annotations to identify first-hand experiences of prescription drug use | Journal of Biomedical Informatics | Wrong study design |
| 85 | Katragadda, Satya; Karnati, Harika; Pusala, Murali; Raghavan, Vijay; Benton, Ryan | Detecting adverse drug effects using link classification on twitter data | Institute of Electrical and Electronics Engineers Inc. | Wrong study design |
| 86 | K. H. U. Chu, J. B.; Allem, J. P.; Pattarroyo, M.; Soto, D.; Cruz, T. B.; Yang, H.; Jiang, L.; Yang, C. C. | Diffusion of Messages from an Electronic Cigarette Brand to Potential Users through Twitter | PLoS ONE [Electronic Resource] | Wrong study design |
| 87 | M. G. P. Kang, H. K.; Choi, S. W.; Kim, H. J.; Kim, J. Y.; Kim, B. K.; Kang, M. K.; Kim, S. H.; Park, H. W.; Cho, S. H.; Min, K. U.; Kim, Y. Y.; Yoon, S. R.; Chang, Y. S. | Discovering the trends in allergic diseases using google trends | Allergy: European Journal of Allergy and Clinical Immunology | Wrong study design |
| 88 | L. N. Shutler, L. S.; Portelli, I.; Blachford, C.; Perrone, J. | Drug Use in the Twittersphere: A Qualitative Contextual Analysis of Tweets About Prescription Drugs | Journal of Addictive Diseases | Wrong study design |
| 89 | P. T. Armenian, S; Gugelmann, H; Gerona, R R | Ease of identifying and purchasing popular “Research Chemicals” via the internet | Clinical Toxicology | Wrong study design |
| 90 | K. H. S. Chu, A. K.; Valente, T. W. | Electronic Cigarette Marketing Online: a Multi-Site, Multi-Product Comparison | JMIR Public Health and Surveillance | Wrong study design |
| 91 | T. M. Katsuki, T K; Cuomo, R | Establishing a Link Between Prescription Drug Abuse and Illicit Online Pharmacies: Analysis of Twitter Data | Journal of Medical Internet Research | Wrong study design |
| 92 | M. G. Chary, N.; Giraud-Carrier, C.; Hanson, C.; Nelson, L.; Manini, A. F. | Estimating nonmedical use of prescription opioids in the USA from social media | Clinical Toxicology | Wrong study design |
| 93 | P. M. Carbonell, M A; Bravo, A | Exploring brand-name drug mentions on Twitter for pharmacovigilance | Studies in Health Technology & Informatics | Wrong study design |
| 94 | Jamnagerwalla, J. Medoff, B. Eilber, K. S. Anger, J. T. | Google trends©: A measure of the societal impact of the FDA transvaginal mesh safety communications | Journal of Urology | Wrong study design |
| 95 | C. B. Adrover, T; Huang, Z; Telenti, A; Salathe, M | Identifying Adverse Effects of HIV Drug Treatment and Associated Sentiments Using Twitter | JMIR Public Health and Surveillance | Wrong study design |
| 96 | G. E. D. Powell, S P; Bell, H G; Anderson, L S; Metcalf, M A | In their own words: Social listening for “real-world benefits” from prescription and OTC products | Value in Health | Wrong study design |
| 97 | M. G. Chary, N.; McKenzie, A.; Manini, A. F. | Leveraging social networks for toxicovigilance | Journal of Medical Toxicology: Official Journal of the American College of Medical Toxicology | Wrong study design |
| 98 | M. T. Thomas, B; Painter, J L; DiSantostefano, R L; Powell, G | Medication discussions on social media-What are people talking about? | Pharmacoepidemiology and Drug Safety | Wrong study design |
| 99 | H. K. A. Yang, S.; Cox, A.; Khankhel, Z.; Martin, A.; Merinopoulou, E. | Methodological issues associated with the use of social media in outcomes research: Case study of adult vaccination | Value in Health | Wrong study design |
| 100 | R. B. L. Correia, L; Rocha, L M | Monitoring Potential Drug Interactions and Reactions Via Network Analysis of Instagram User Timelines | Pacific Symposium on Biocomputing | Wrong study design |
| 101 | V. S. Dimov, M.; Casale, T. | North-South gradient in online search volumes for allergy-related queries in the United States may suggest a potential role of vitamin D | Journal of Allergy and Clinical Immunology | Wrong study design |
| 102 | B. M. A. Martinez, C. V.; Dailey, F.; Desai, M. P.; Dupuy, T.; Mosadeghi, S.; Whitman, C. B.; Lasch, K.; Ursos, L.; Spiegel, B. | Patient understanding of the risks and benefits of biologic therapies in inflammatory bowel disease (IBD): Insights from a large-scale analysis of social media platforms | Gastroenterology | Wrong study design |
| 103 | J. D. Tyrawski, D C | Pharmaceutical companies and their drugs on social media: a content analysis of drug information on popular social media sites | Journal of Medical Internet Research | Wrong study design |
| 104 | H. G. S. Bell, L; Rodriguez, H; Pierce, C; Dasgupta, N; Shaikh, S; Powell, G E | Pharmaceutical products and vaccines discussed in social media: Which ones are patients talking about? | Value in Health | Wrong study design |
| 105 | K. P. O'Connor, P.; Nikfarjam, A.; Ginn, R.; Smith, K. L.; Gonzalez, G. | Pharmacovigilance on twitter? Mining tweets for adverse drug reactions | AMIA ... Annual Symposium Proceedings/AMIA Symposium | Wrong study design |
| 106 | L. P. Shutler, J.; Portelli, I.; Nelson, L. S.; Blachford, C. R. | Prescription opioids in the twittersphere: A contextual analysis of tweets about prescription drugs | Annals of Emergency Medicine | Wrong study design |
| 107 | L. R. Thompson, F. P.; Whitehill, J. M. | Prevalence of Marijuana-Related Traffic on Twitter, 2012-2013: A Content Analysis | Cyberpsychology, behavior and social networking | Wrong study design |
| 108 | R. L. P. DiSantostefano, J. L.; Thomas, M.; Powell, G. | Safety assessment and selection bias: Who uses social media to communicate about medications? | Pharmacoepidemiology and Drug Safety | Wrong study design |
| 109 | L. B. Schifano, H. G.; Anderson, L. S.; Shaikh, S.; Powell, G. E. | Social media data shows utility for the assessment of benefit-risk early after prescription to over-the-counter switch | Value in Health | Wrong study design |
| 110 | G. E. S. Powell, H. A.; Reblin, T.; Burstein, P. J.; Blowers, J.; Menius, J. A.; Painter, J. L.; Thomas, M.; Pierce, C. E.; Rodriguez, H. W.; Brownstein, J. S.; Freifeld, C. C.; Bell, H. G.; Dasgupta, N. | Social Media Listening for Routine Post-Marketing Safety Surveillance | Drug Safety | Wrong study design |
| 111 | A. O. C. Sarker, K.; Ginn, R.; Scotch, M.; Smith, K.; Malone, D.; Gonzalez, G. | Social Media Mining for Toxicovigilance: Automatic Monitoring of Prescription Medication Abuse from Twitter | Drug Safety | Wrong study design |
| 112 | B. L. Chan, A.; Sarkar, U. | The Canary in the Coal Mine Tweets: Social Media Reveals Public Perceptions of Non-Medical Use of Opioids | PLoS ONE [Electronic Resource] | Wrong study design |
| 113 | C. L. B. Hanson, S. H.; Giraud-Carrier, C.; West, J. H.; Barnes, M. D.; Hansen, B. | Tweaking and tweeting: exploring Twitter for nonmedical use of a psychostimulant drug (Adderall) among college students | Journal of Medical Internet Research | Wrong study design |
| 114 | G. M. Furgiuele, M; Smith, D C; Love, B L; Bookstaver, P B; Sides, A | Twitter evaluation and extension of Pharmacy study (TWEEPS): An evaluation of hydrocodone | Pharmacotherapy | Wrong study design |
| 115 | Andrew McNeill , Peter R. Harris and Pam Briggs | Twitter influence on UK Vaccination and antiviral Uptake during the 2009 h1n1 Pandemic | Frontiers in Public Health | Wrong study design |
| 116 | M. P. Roccetti, C.; Salomoni, P.; Marfia, G. | Unleashing the true potential of social networks: confirming infliximab medical trials through Facebook posts | Network Modeling and Analysis in Health Informatics and Bioinformatics | Wrong study design |
| 117 | S. W. Ramagopalan, R.; Cox, A. P. | Using Twitter to investigate opinions about multiple sclerosis treatments: a descriptive, exploratory study | F1000Research | Wrong study design |
| 118 | H. G. A. Bell, L S; Yuen, N A; Ruane, B; Seifert, H; Powell, G E | Utility of social listening in pharmacovigilance for groups of special interest: Product complaints, pregnancy, pediatric, and the elderly | Pharmacoepidemiology and Drug Safety | Wrong study design |
| 119 | Somrak Numnark | VaccineWatch : a monitoring system of vaccine messages from social media data | he 8th International Conference on Systems Biology (ISB) | Wrong study design |
| 120 | Risson, V.Saini, D.Bonzani, I.Huisman, A.Olson, M. | Validation of social media analysis for outcomes research: Identification of drivers of switches between oral and injectable therapies for multiple sclerosis | Value in Health | Wrong study design |
| 121 | T. T. Trenque, M A; De Boissieu, P; Herlem, E | Web traffic: A new tool in pharmacovigilance? | Pharmacoepidemiology and Drug Safety | Wrong study design |
| 122 | O. J. C.-S. Dyar, E.; Holmes, A. H. | What makes people talk about antibiotics on social media? A retrospective analysis of Twitter use | Journal of Antimicrobial Chemotherapy | Wrong study design |
| 123 | Zhang, T.; Lin, H.; Ren, Y.; Yang, L.; Xu, B.; Yang, Z.; Wang, J.; Zhang, Y. | Adverse drug reaction detection via a multihop self-attention mechanism | | Wrong study design |
