# Supplementary material for: Social media and internet search data to inform drug utilization: A systematic scoping review
Source: Front Digit Health. 2023 Mar 20;5:1074961. doi: 10.3389/fdgth.2023.1074961 (PMC10067924; doi:10.3389/fdgth.2023.1074961)
Supplement: Supplementary file 4 [file Datasheet4.pdf]

Supplementary Material (File S4): Characteristics of included studies (Ordered by year of publication) Keller et al. 2023

General study overview

Study ID	Country of correspon-ding author	Study design	Objective	Medication of interest	Statistics of comparison	Main findings (extensive)
Schuster *et al.* (2010)	US	Retrospective longitudinal study	To evaluate the association of the relative search volume of the search term *Lipitor* with Lipitor global revenues.	Lipitor (atorvastatin calcium), simvastatin (Zocor)	Quantitative, Pearson correlation coefficient	- The mean number of Google search queries for Lipitor significantly decreased (−0.00323 slope), while the queries for simvastatin increased (0.00176 slope) from January 2004 to June 2009 ($P < 0.001$ for both).<br>- The percentage change in annual Lipitor global revenues decreased from 18% in 2004 to 2 % in 2008 and significantly correlated ($r = 0.98$, $p < 0.001$) with the mean Google query index for Lipitor which decreased during the same period.<br>*- This study included additional analyses regarding the community-based resource use per Medicare beneficiary that are out of scope for this review
Simmering *et al.* (2014)	US	Retrospective longitudinal study	To evaluate the association between drug utilization estimates of several seasonal prescription drugs and the corresponding Google Trends search volume.	Amoxicillin, azelastine, azithromycin, benzonatate, cefdinir, ciprofloxacin, levofloxacin, moxifloxacin, olopatadine.	Quantitative, Cross-correlation function	- Only three out of nine seasonal drugs considered had enough outpatient dispensing events in the MEPS data to construct a time series suitable for analysis (amoxicillin, azithromycin and cefdinir). These 3 drugs showed positive correlation between the search volume and drug utilization estimates at lags near 0 (i.e. in the same week).<br>- A strong positive relationship between drug utilization estimates and search volumes was also detected at year intervals and a strong negative relationship at half-year intervals.<br>*- This study includes additional analyses about knowledge events that are out of scope for this review
Skeldon *et al.* (2014)	Canada	Ecologic analysis	To evaluate the association of two direct-to-consumer advertising (DTCA) campaigns with the volume of Internet searches for "Avodart" (dutasteride) and "Flomax" (tamsulosin) and to evaluate the association of the DTCA campaigns with the prescription rates of dutasteride and tamsulosin.	Dutasteride (Avodart®), tamsulosin (Flomax®)	No direct comparison but parallel reporting of results both data sources	- The dutasteride campaign was significantly correlated with increases in search volumes for "Avodart" (level change +31.3 %, 95 % CI: 27.2–35.4) and "Flomax" (level change +8.3 %, 95 % CI: 0.9–15.7) and with increases in the prescription of dutasteride (trend = 0.45/month, 95 % CI: 0.33–0.56) and tamsulosin (trend = 0.76/month, 95 % CI: 0.02–1.50).<br>- The tamsulosin campaign was significantly associated with increased "Flomax" search volumes (level change +25.3 %, 95 % CI: 18.7–31.8) and with immediate increases in the prescription of dutasteride (level change +1.47 units, 95 % CI: 0.79–2.14) and tamsulosin (level change +5.76 units, 95 % CI: 1.79–9.72).
Gahr *et al.* (2015)	Germany	Retrospective longitudinal study	To evaluate the association of annual prescription volumes of several antidepressants with marketing approval in Germany with corresponding Google Trends web search query volumes.	"Valdoxan" (~agomelatine), "Elontril" (~bupropion), "Citalopram" (~citalopram), "Cipralex" (~escitalopram), "Fluoxetin" (~fluoxetine), "Fluvoxamine (~fluvoxamine), "Paroxetin" (~paroxetine), "Sertralin" (~sertraline)	Quantitative, Pearson's r *Interpreted by the review authors as Pearson's r and not Person's r must have been meant as stated in the paper.	Significant and strong correlations between substance-specific annual prescription volumes and corresponding annual query volumes were found for each substance during the observational interval: (agomelatine: $r = 0.968$, $R^2 = 0.932$; bupropion: $r = 0.962$, $R^2 = 0.925$, citalopram: $r = 0.970$, $R^2 = 0.941$, escitalopram: $r = 0.824$, $R^2 = 0.682$, fluoxetine: $r = 0.885$, $R^2 = 0.783$, paroxetine: $r = 0.801$, $R^2 = 0.641$, sertraline: $r = 0.880$, $R^2 = 0.689$; $p = 0.01$ for all correlations).
Jha *et al.* (2015)	US	Ecological analysis	To investigate trends in media reports and public interest of bisphosphonates using Google Trends as well as to estimate the trends in oral bisphosphonate use among patients aged ≥55 years using national health survey data.	Oral bisphosphonates	No direct comparison but parallel reporting of results both data sources	- A series of spikes in search volume for "Fosamax" (alendronate) occurred between 2006 and 2010 immediately following media reports of safety concerns.<br>- The prevalence of oral bisphosphonate use declined by greater than 50% between 2008 and 2012 ($p < 0.001$) after increasing use for more than a decade.<br>*- This study included additional analyses regarding the incidence and hospitalization rates of intertrochanteric and subtrochanteric fractures that are out of scope for this review
Kalichman *et al.* (2015)	US	Retrospective longitudinal study with cross-sectional comparison	To examine the associations of the internet search activity for H1N1 and human papilloma virus (HPV) disease and vaccine information with H1N1 and HPV vaccine uptake.	H1N1 flu vaccine, HPV vaccine	Quantitative, Spearman's rho correlation and ordinal regression analysis for multivariable models	- The search term H1N1 peaked in October, whereas HPV Internet searches were not seasonal. (not reported seasonality for comparison data source)<br>- The search term H1N1 significantly correlated with all target groups with rho ranging from 0.45 to 0.57. The search term vaccine significantly correlated with vaccine coverage for all age groups younger than 65 years, with rho ranging from 0.32 to 0.49. For persons older than 65 years, the correlation was not significant, rho = 0.22. Ordinal regression showed that the H1N1 search term was independently associated with H1N1 vaccine coverage, Wald $\chi 2 = 10.41$, $p < 0.001$.<br>- Similarly, the correlation between the search volume of the term vaccine and HPV coverage was significant (rho = 0.47, $p < 0.01$). Ordinal regression found that vaccine search volume independently predicted HPV coverage, Wald $\chi 2 = 5.39$, $p < 0.05$.
Crowson *et al.* (2016)	US	Retrospective longitudinal study	To evaluate common ototopical antibiotics' prescription volumes association with corresponding Google Trends search volumes and to investigate the seasonality of national prescription volumes and Google Trends search volume.	Ciprofloxacin-dexamethasone,'' ''Cortisporin,'' ''Ofloxacin,''	Quantitative, Pearson's correlation coefficient	- Google Trends user search interest showed significant correlations to Medicaid prescription volumes for Ciprofloxacin-dexamethasone ($r = 0.38$, $p = 0.046$), Ofloxacin ($r = 0.74$, $p < 0.001$), Cortisporin ($r = 0.49$, $p = 0.008$).<br>- Google Trends user search interest showed analogous sinusoidal seasonality to Medicaid prescription data with annual peaks in the summer months of June to September.
Hansen *et al.* (2016)	Denmark	Proof-of-concept of prediction models	To develop and evaluate prediction models using clinical and web-mined data for predictions about future vaccination uptake for all official recommended children Vaccines in Denmark.	All official children Vaccines in Denmark: DiTeKiPol-1, DiTeKiPol-2, DiTeKiPol-3, DiTeKiPol-4, PCV-1, PCV-2, PCV-3, MMR-1, MMR-2(4), MR-2(12), HPV-1, HPV-2 and HPV-3	Qualitative, root mean squared error	- For 10/13 officially recommended children vaccines in Denmark the ensemble learning method that combined web and clinical data for prediction outperformed predictions using either clinical or web data alone.<br>- Using only web data gives predictions with an overall error only slightly worse than for the predictions made using only clinical data.
Jankowski *et al.* (2016)	Poland	Retrospective longitudinal study cross-sectional comparison	To develop a method using Google search engine data to rank psychoactive drugs according to their popularity and to qualitatively compare the popularity ranking to international drug report data.	Alcohol, amphetamine, benzodiazepines, buprenorphine, butane, cannabis, cocaine, ecstasy, gamma-hydroxybutyric acid (GHB), heroin, ketamine, khat, lysergic acid diethylamide (LSD), mephedrone, methadone, methamphetamine	Qualitative, Popularity ranking list	- Alcohol was found to be the most popular psychoactive drug with a relative popularity index of 100%, followed by cannabis, 15.2%; cocaine, 15.1%; LSD, 12.5%; heroin, 12.0; ecstasy, 11.0%; GHB, 6.0%; methadone, 3.4%; butane, 3.0%; khat, 2.7%; amphetamine, 2.3%; methamphetamine, 2.3%; ketamine, 2.2%; buprenorphine, 1.6%; benzodiazepines, 1.2%; and mephedrone, 0.5%.<br>- The popularity ranking correlated with the UNODC report data of 2011, where after amphetamine-type stimulants (ecstasy, amphetamine, and methamphetamine) the most seized drugs were cannabis, cocaine, LSD and heroin.<br>- Except LSD, the popularity ranking were also quite similar to the European Drug Report 2014: Trends and Developments that shows cannabis as the most frequently seized illegal drug, before cocaine, heroine, ecstasy, amphetamine, methamphetamine, and LSD.<br>*- This study included additional analyses about the harmfulness of drugs that are out of scope for this review

Song *et al.* (2017)	US	Retrospective longitudinal study	To develop a method using Twitter data for flu vaccination monitoring and to evaluate the method against official flu vaccination surveillance data.	Influenza vaccination	Quantitative, Pearson correlation coefficient	Correlation coefficients between 0.876 and 0.997 and p-values of less than 0.00001 indicate a significant, positive linear relationship between the number of twitter posts and flu vaccination immunization rates.
Hansen *et al.* (2018)	Denmark	Proof-of-concept of prediction models	To develop and evaluate prediction models using web search and antimicrobial purchase data for predictions about future antimicrobial drug consumption.	Antibiotics; subgroup: beta-lactamase sensitive penicillins (J01CE)	Qualitative , root mean squared error and mean absolute error	- Overall, the use of web data only gives predictions that are slightly more erroneous, but generally not that far off, from those made when using only historical antimicrobial purchase data.<br>- Best predictions were found when combining both web search and purchase data.
Huang *et al.* (2018)	US	Cross-sectional study	To develop a method based on a machine learning classifier that employs Twitter data for real-time influenza vaccination surveillance and to evaluate the method by comparing to published government survey data.	Influenza vaccination	Quantitative, Pearson correlation coefficient	- Both data sources show seasonal peaks in October, when influenza vaccines are distributed in the USA<br>- Correlations of 0.799 (95%-CI: 0.797 to 0.801) between monthly Twitter estimates and governmental data were found, with geographical correlations of 0.387 (95%-CI: 0.362 to 0.394) at US state level and 0.467 (95%-CI: 0.445 to 0.483) at the regional level.<br>- More tweets were found for female twitter users compared to male users, consistent with the results of the Centers for Disease Control and Prevention on vaccine uptake.
Kamiński *et al.* (2019)	Poland	Retrospective longitudinal study with cross-sectional comparison	To analyse the association of the Google Trends' relative search volume for the topics antibiotics and probiotics with antibiotic consumption worldwide.	Antibiotics, probiotics	Quantitative, Spearman rank-correlation	'- The mean relative search volume (RSV) of antibiotics was equal to 57.7 ± 17.9, rising by 3.7 RSV/year (6.5%/year) and probiotic relative search volume was equal to 14.1 ± 7.9, rising by 1.7 RSV/year (12.1%/year).<br>- Antibiotic consumption was significantly associated with the relative search volume of probiotics (Rs = 0.35; p < 0.01), but not with antibiotics (Rs = 0.14; p > 0.05).<br>- The seasonal peaks of the relative search volume for both probiotics and antibiotics were observed in the cold months, and the seasonal amplitude was equal to a mean relative search volume of 9.8 for antibiotics and 2.7 for probiotics.<br>*- This study included additional analyses regarding the association between antibiotic and probiotic search volumes with health expenditure per capita, the 2015 Human Development Index and the 2015 drug resistance index that are out of scope for this review.
Mimura *et al.* (2019)	Japan	Retrospective Observational Study	To examine prescription trends in heparinoid (moisturizer) use and analyse their association with Google Trends search volume.	Heparinoid	Quantitative, Cross-correlation	- The number of heparinoid prescriptions increased from 2011 onwards<br>- The number of internet searches increased from 2012 onwards<br>- Internet searches were significantly correlated with total heparinoid prescription (correlation coefficient = 0.25, P = 0.005).<br>- Internet searches were significantly correlated with heparinoid prescription in the age group of 20-59 years at –1-month lag in Google Trends (correlation coefficient = 0.30, P = 0.001).

Data source characteristics

Study ID	Comparative data source, measure in data source, location of data origin	Web data source, measure in data source, location of data origin	Accessed time period of comparison data source	Accessed time period of web data source	Total duration comparison data source was accessed (years)	Total duration web data source was accessed (years)
Schuster *et al.* (2010)	Pfizer Annual Shareholder Reports 2004 - 2008, Global revenues, worldwide	Google Trends/Google Insight for Se	years: 2004-2008	4 January 2004 - 28 June 2009	5	5.5
Simmering *et al.* (2014)	Medical Expenditure Panel Survey (MEPS), Drug utilization estimates, USA	Google Trends, search volume	2004-2009	n/a	5	n/a
Skeldon *et al.* (2014)	IMS Health, Prescription rates, USA	Google trends, search volume, USA	January 2003 - December 2007	January 2003 - December 2007	5	5
Gahr *et al.* (2015)	"Arzneiverordnungs-Report", Prescription volumes, Germany	Google Trends, search term frequen	2005-2014	2004-2013	9	9
Jha *et al.* (2015)	The Medical Expenditure Panel Survey (MEPS), Estimation of medication utilization based on prescription volumes, USA	Google Trends, search volume, USA	1996- 2012	January 2004 - January 2015	16	11
Kalichman *et al.* (2015)	Centers for Disease Control and Prevention, Vaccination coverage, USA	Google Insight for Search, query ind	H1N1: peak flu season of 2009; HPV: 2010, but period unclearly stated	H1N1: peak flu season of 2009; HPV: 2010	1	1
Crowson *et al.* (2016)	Medicaid, Prescription volumes, USA	Google Trends, search volume, USA	January 2008 - July 2014	January 2008 - July 2015	6.5	7.5
Hansen *et al.* (2016)	State Serum Institut, Vaccination uptake, Denmark	Google trend, search queries, Denm	January 2011 - September 2015	January 2011 - September 2015	4.75	4.75

Jankowski *et al.* (2016)	UNODC World Drug Report from 2011 and European Drug Report 2014: Trends and Developments, Number of drug seizures, worldwide (UNDOC World Drug Report) and European Union, Turkey, Norway (European Drug Report)	Google search engine, frequency of	UNODC drug report 2011: Year 2010<br><br>European Drug Report 2014: Trends and Developments: 2012 or the most recent year available (before 2012)	June 20, 2014 with data available before May 1, 2012, October 1, 2012, January 1, 2013, July 1, 2013, and February 1, 2014.	n/a	n/a
Song *et al.* (2017)	Flu vaccination rate surveillance system used by the United States Department of Health and Human Services, Immunization rates of flu vaccination, USA	Twitter, number of twitter posts, US	13 June 2013- 26 May 2017	11 August 2012 - 26 May 2017	3.9	4.75
Hansen *et al.* (2018)	Register of Medicinal Product Statistics, Sales of antimicrobials, Denmark	Google Health Trends, search query	January 2007- 23 October 2016	2 January 2011 - 23 October 2016	9.83	5.83
Huang *et al.* (2018)	Centers for Disease Control and Prevention 's FluVaxView system, Influenza vaccination activity data, USA	Twitter, number of twitter posts, US	July 2013 - May 2017 (excluding month of June), but period unclearly stated	July 2013 - May 2017 (excluding month of June)	3.67	3.67
Kamiński *et al.* (2019)	The Center for Disease Dynamics Economics & Policy, Antibiotic consumption, worldwide	Google Trends, relative search volu	Year 2015	For time series: January 2004 to 7 June 2019<br><br>For correlation: Year 2015	1	1
Mimura *et al.* (2019)	Administrative claims database provided by JMDC Inc, Prescription volume, Japan	Google trends, search volume, Japa	October 1, 2007 to September 31, 2017	October 1, 2007 to September 31, 2017	10	10

Additional study items

Study ID	Funding (yes/no)	Funding which?	Conflict of interest	Which conflict of interest?	limitations described	Reference	Journal/conference/workshop
Schuster *et al.* (2010)	None reporte	n/a	no	n/a	- Query data cannot be used to establish causation, as patients may search for the drug before or after the physician describes it.<br>- Search intend of user should be taken into account for searches that were created not due to the behaviour of interest. For example, patients might not only search for a drug after the physician describes it but also after a new study involving that drug receives media coverage.	Nathaniel M. Schuster, BS; Mary A.M. Rogers, PhD, MS; and Laurence F. McMahon Jr, MD, MPH;<br>Using Search Engine Query Data to Track Pharmaceutical Utilization: A Study of Statins;<br>AJMC<br>2010	AJMC 2010 (American Journal of Managed Care)
Simmering *et al.* (2014)	No	n/a	Nothing stated	n/a	- The elderly are the largest consumers of medications and also underrepresented among users of search engines resulting in a potential mismatch between the users of the medications and those generating the search data.<br>- Google Trends only reports a normalized share which makes conversion to and absolute scale difficult, as the same number of total searches at different times may have two different volume estimates.<br>- MEPS data may have high inter-week variance which makes it difficult to construct meaningful time for weeks with only little amount of fills<br>- MEPS data might not capture all drug counts if the pharmacy reported obscure names that did not contain elements of the generic names or typical brand names.	Jacob E. Simmering, M.S.a<br>, Linnea A. Polgreen, Ph.D.a,<br>Philip M. Polgreen, M.D., M.P.H.<br>"Web search query volume as a measure of pharmaceutical utilization and changes in prescribing patterns"<br>Research in social and Administrative Pharmacy;<br>2014<br>volume 10<br>page 896-903	Research in social and Administrative Pharmacy;
Skeldon *et al.* (2014)	Yes	Sean Skeldon: funds from the Eunice Kennedy Shriver National Institute of Child Health and Human Development, the Office of Research on Women's Health, and the National Institute on Aging, at the National Institutes of Health, administered by the University of Minnesota Deborah E. Powell Center for Women's Health.	no	n/a	- Observational study of ecologic data cannot definitively conclude that there is a causal relationship between the DTCA campaigns and changes in Internet search and dispensed prescription levels.<br>- Difficult to assess whether physicians themselves were influenced by DTCA itself rather than by patient requests.<br>- Tamsulosin was approved four years before the approval of dutasteride. However, it is not possible to determine whether the results would be similar if the order of the campaigns were reversed.<br>- The study focused on a single disease involving men, and thus the results may not be more broadly generalizable.	Skeldon SC, Kozhimannil KB, Majumdar SR, Law MR. The Effect of Competing Direct-to-Consumer Advertising Campaigns on the Use of Drugs for Benign Prostatic Hyperplasia: Time Series Analysis. J Gen Intern Med. 2014;30:514–20.	Journal of internal Medicine

Gahr *et al.* (2015)	No	n/a	no	n/a	- "Arzneimittelverordnungsreport" only provides substance-specific prescription volumes relating to a long (annual) period. Therefore, it remains unsettled whether demonstrated correlations are also detectable for shorter periods<br>- Trend of parallel increase in all substance-specific datasets of web and comparison data source over time suggests the possibility of a cohort effect<br>- Elderly people are the largest population using pharmaceuticals but underrepresented internet users.<br>- Population who has generated web data cannot be addressed sufficiently with chosen approach<br>- Found relations are still undetermined for pharmacological agents other than antidepressants and countries other than Germany.	M. U. Gahr, Z; Zeiss, R; Connemann, B J; Lang, D; Schönfeldt-Lecuona, C .<br>Linking Annual Prescription Volume of Antidepressants to Corresponding Web Search Query Data.<br>Journal of Clinical Psychopharmacology.<br>Volume 35,<br>Number 6,<br>December 2015	Journal of Clinical Psychopharmacology.
Jha *et al.* (2015)	Yes	Research supported by the Intramural Research Program of the National Institute of Arthritis and Musculoskeletal and Skin Diseases (NIAMS) of the National Institutes of Health(NIH).	no	n/a	Not reported	Jha S, Wang Z, Laucis N, Bhattacharyya T. Trends in Media Reports, Oral Bisphosphonate Prescriptions, and Hip Fractures 1996-2012: An Ecological Analysis. J Bone Miner Res. 2015;30(12):2179–87.	Journal of Bone and Mineral Research
Kalichman *et al.* (2015)	Yes	Research supported by a Grand Challenges Exploration Grant from the Bill and Melinda Gates Foundation	Nothing stated	n/a	- Associations between internet search activity and vaccination uptake cannot be interpreted as causal relationships<br>- Unmeasured factors that may account for both increased vaccination and internet searches: a state's socioeconomic conditions, immunization policies, investments in vaccination campaigns, individuals' attitudes towards vaccination	S. C. Kalichman and C. Kegler; Vaccine-Related Internet Search Activity Predicts<br>H1N1 and HPV Vaccine Coverage: Implications for<br>Vaccine Acceptance;<br>Journal of Health Communication;<br>2017	Journal of Health Communication;
Crowson *et al.* (2016)	No	n/a	Nothing stated	n/a	- Google Trends does not make volume of user search terms public which limits the ability to infer associations between user search and prescription frequency to general upward and downward trends.<br>- Types of users (eg providers, patients, general public) cannot be differentiated using Google Trends data<br>- Medicaid data does not include prescriptions for patients who do not meet low-income inclusion criteria or third-party payers.	Crowson, M G; Schulz, K; Tucci, D L<br>National Utilization and Forecasting of Ototopical Antibiotics: Medicaid Data Versus "Dr. Google"<br>Otology & Neurotology<br>2016<br>Volume:23<br>Pages: 23	Otology & Neurotology<br>2016
Hansen *et al.* (2016)	None reported	n/a	Nothing stated	n/a	Not reported	Niels Dalum Hansen, Christina Lioma, and Kåre Mølbak. 2016. Ensemble Learned Vaccination Uptake Prediction using Web Search Queries. In Proceedings of the 25th ACM International on Conference on Information and Knowledge Management (CIKM '16). Association for Computing Machinery, New York, NY, USA, 1953–1956. DOI:https://doi.org/10.1145/2983323.2983882	Proceedings of the 25th ACM International on Conference on Information and Knowledge Management (CIKM '16).
Jankowski *et al.* (2016)	Yes	Research supported in part by PL-Grid Infrastructure	no	n/a	Not reported	W. H. Jankowski, M<br>Can Google Searches Predict the Popularity and Harm of Psychoactive Agents?<br>2016<br>JMIR Publications	JMIR Publications
Song *et al.* (2017)	Yes	By National Science Foundation (SNF) and the United States Department of Defense	Nothing stated	n/a	- Twitter data relies on self-reported experiences and therefore might be unreliable<br>- Younger Americans of 18-29 years are disproportionately represented on twitter, so data might not accurately reflect general population vaccination rates	S. Song and Z. B. Miled, "Digital Immunization Surveillance: Monitoring Flu Vaccination Rates Using Online Social Networks," 2017 IEEE 14th International Conference on Mobile Ad Hoc and Sensor Systems (MASS), Orlando, FL, 2017, pp. 560-564. doi: 10.1109/MASS.2017.96	2017 IEEE 14th International Conference on Mobile Ad Hoc and Sensor Systems (MASS)
Hansen *et al.* (2018)	None reported	n/a	No	n/a	Not reported	Hansen ND, Mølbak K, Cox I, Lioma C. Predicting antimicrobial drug consumption using web search data. ACM Int Conf Proceeding Ser. 2018;2018-April:133–42.	DH '18: Proceedings of the 2018 International Conference on Digital Health
Huang *et al.* (2018)	Yes	Manuscript Preparation was supported by the National Institute of General Medical Sciences and by the National Science Foundation	Yes	Two authors (MD and MJP) hold equity in Sickweather Inc. MD has received consulting fees from Bloomberg LP, and holds equity in Good Analytics Inc. These organisations did not have any role in the study design, data collection and analysis, decision to publish or preparation of the manuscript.	- While Twitter can be considered 'big data', the sample size is more limited when narrowed to specific populations<br>- Certain vulnerable populations, including children and older adults, are underrepresented in Twitter data	Huang X, Smith MC, Jamison AM, et al. Can online self-reports assist in real-time identification of influenza vaccination uptake? A cross-sectional study of influenza<br>vaccine-related tweets in the USA, 2013–2017. BMJ Open 2018;9:e024018. doi:10.1136/<br>bmjopen-2018-024018	bmjopen-2018
Kamiński *et al.* (2019)	No	n/a	Yes	Two authors are the foundation shareholders of Sanprobi, the manufacturer and distributor of the probiotics. One author received remuneration from this company, and the content of this study was not subjected to any constraints by this company	- Google Trends only provides estimation of the relative search volume, but it is not possible to assess a precise number of queries<br>- The relative search volume of Google Trends could be dependent on media attention<br>- Results are limited because of low search volume in many, mostly African countries<br>- Because of limited data on antibiotic consumption, only correlation test for 2015 could be performed	Kamiński M, Łoniewski I, Marlicz W. Global Internet Data on the Interest in Antibiotics and Probiotics Generated by Google Trends. Antibiotics. 2019;8(3):147.	Antibiotics 2019
Mimura *et al.* (2019)	None reported	n/a	No	n/a	- Data from employees of small and medium sized business, public officials, self-employed people and their families are underrepresented in JMDC database. Therefore, results cannot be generalised to a wider population in Japan.<br>- Google and Yahoo are main internet search engines in Japan, and Google Trends does not include entire Japanese population<br>- Google Trends gives only information about search queries, but does not provide access to details about how research words were recognized and aggregated on google<br>- Study only examined associations between internet searches and prescriptions. Therefore, study did not clarify cause of the increase in prescriptions or the number of people prescribed the moisturizer for cosmetic purposes due to lack of information on attitudes and prescription behaviours.	Mimura, Wataru & Akazawa, Manabu. (2018). Association between Internet searches and moisturizer prescription in Japan (Preprint). 10.2196/preprints.13212.	JMIR (Journal of Medical Internet Research) Public Health & Surveillance

Supplementary Material (File S4): Reporting of items of the STROBE statement (Strengthening the Reporting of Observational Studies in Epidemiology) comple-mented with items from the RECORD and RECORD-PE checklists (Reporting of studies conducted using observational routinely collected data (RECORD) and RECORD statement for pharmacoepidemiological research (RECORD-PE))
Keller et al. 2023
Abbreviations: + = item fulfilled, p = item partially fulfilled, - = item not fulfilled, n/a = item not applicable

Item Nr:	STROBE	RECORD	RECORD-PE	Item Category	Item description	Schuster *et al.* (2010)	Simmering *et al.* (2014)	Skeldon *et al.* (2014)	Gahr *et al.* (2015)	Jha *et al.* (2015)	Kalichman *et al.* (2015)	Crowson *et al.* (2016)	Hansen *et al.* (2016)	Jankowski *et al.* (2016)	Song *et al.* (2017)	Hansen *et al.* (2018)	Huang *et al.* (2018)	Kamiński *et al.* (2019)	Mimura *et al.* (2019)	Total Yes (n, %)		Partly (n, %)		No (n, %)		not appl. (n, %)	
Title and abstract																											
1	(a)				Indicate the study's design with a commonly used term in the title or the abstract.	+	-	p	-	+	-	-	-	-	-	-	+	-	+	4	(29)	1	(7)	9	(64)	0	(0)
	(b)				Provide in the abstract an informative and balanced summary of what was done and what was found.	+	+	+	+	+	+	p	p	+	p	+	+	+	+	11	(79)	3	(21)	0	(0)	0	(0)
		1.1			The type of data used should be specified in the title or abstract. When possible, the name of the databases used should be included.	+	+	+	+	+	+	+	p	+	+	+	+	+	+	13	(93)	1	(7)	0	(0)	0	(0)
		1.2			If applicable, the geographical region and timeframe within which the study took place should be reported in the title or abstract.	+	-	+	+	+	+	p	-	-	-	p	+	p	+	7	(50)	3	(21)	4	(29)	0	(0)
Introduction																											
2				Background/rationale	Explain the scientific background and rationale for the investigation being reported.	+	+	+	+	+	+	+	+	+	+	+	+	+	+	14	(100)	0	(0)	0	(0)	0	(0)
3				Objectives	State specific objectives, including any prespecified hypotheses.	+	+	+	+	+	+	+	+	p	+	+	+	+	+	13	(93)	1	(7)	0	(0)	0	(0)
Methods																											
4				Study design	Present key elements of study design early in the paper.	+	+	p	+	+	+	+	p	+	+	p	+	+	+	11	(79)	3	(21)	0	(0)	0	(0)
5				Setting	Describe the setting, locations, and relevant dates, including periods of recruitment, exposure, follow-up, and data collection.	+	p	+	+	+	+	p	+	p	+	+	+	+	+	11	(79)	3	(21)	0	(0)	0	(0)
		7.1		Variables	A complete list of codes and algorithms used to classify exposures, outcomes, confounders, and effect modifiers should be provided. If these cannot be reported, an explanation should be provided.	-	-	-	-	p	-	-	-	-	-	p	-	-	+	1	(7)	2	(14)	11	(79)	0	(0)
			7.1.a		Describe how the drug exposure definition was developed.	n/a	n/a	n/a	n/a	n/a	n/a	n/a	n/a	n/a	n/a	n/a	n/a	n/a	n/a	0	(0)	0	(0)	0	(0)	14	(100)
			7.1.b		Specify the data sources from which drug exposure information for individuals was obtained.	+	+	+	+	+	+	+	+	+	+	+	+	+	+	14	(100)	0	(0)	0	(0)	0	(0)
9				Bias	Describe any efforts to address potential sources of bias.	-	-	-	-	-	+	-	-	-	-	-	-	-	-	1	(7)	0	(0)	13	(93)	0	(0)
12	(a)			Statistical methods	Describe all statistical methods, including those used to control for confounding.	+	+	+	+	+	+	+	+	p	+	+	+	+	+	13	(93)	1	(7)	0	(0)	0	(0)
	(e)				Describe any sensitivity analyses.	n/a	n/a	n/a	n/a	+	n/a	n/a	n/a	n/a	n/a	n/a	n/a	n/a	n/a	1	(7)	0	(0)	0	(0)	13	(93)
12		12.1		Data access	Authors should describe the extent to which the investigators had access to the database.	+	+	+	+	+	p	+	p	p	+	+	+	p	+	10	(71)	4	(29)	0	(0)	0	(0)
Results																											
13	(c)			Participants	Consider use of a flow diagram.	n/a	n/a	n/a	n/a	n/a	n/a	n/a	n/a	n/a	n/a	n/a	n/a	n/a	+	1	(7)	0	(0)	0	(0)	13	(93)
15				Outcome data	Cohort study—report numbers of outcome events or summary measures over time. Case-control study—report numbers in each exposure category, or summary measures of exposure. Cross sectional study—report numbers of outcome events or summary measures.	+	p	+	+	+	+	+	+	+	+	+	+	+	+	13	(93)	1	(7)	0	(0)	0	(0)
17				Other analyses	Report other analyses done—eg, analyses of subgroups and interactions, and sensitivity analyses.	n/a	n/a	n/a	n/a	+	n/a	n/a	n/a	n/a	n/a	n/a	n/a	n/a	n/a	1	(7)	0	(0)	0	(0)	13	(93)
Discussion																											
18				Key results	Summarise key results with reference to study objectives.	p	p	-	-	-	+	p	n/a	-	n/a	n/a	+	+	+	4	(29)	3	(21)	4	(29)	3	(21)
19				Limitations	Discuss limitations of the study, taking into account sources of potential bias or imprecision. Discuss both direction and magnitude of any potential bias.	+	+	+	+	+	+	+	-	-	+	-	+	+	+	11	(79)	0	(0)	3	(21)	0	(0)
		19.1			Discuss the implications of using data that were not created or collected to answer the specific research question(s). Include discussion of misclassification bias, unmeasured confounding, missing data, and changing eligibility over time, as they pertain to the study being reported.	+	+	+	+	+	p	+	-	-	+	-	p	p	+	8	(57)	3	(21)	3	(21)	0	(0)
			19.1.a		Describe the degree to which the chosen database(s) adequately captures the drug exposure(s) of interest.	p	p	+	p	p	p	+	-	-	+	-	+	-	+	5	(36)	5	(36)	4	(29)	0	(0)
20				Interpretation	Give a cautious overall interpretation of results considering objectives, limitations, multiplicity of analyses, results from similar studies, and other relevant evidence.	+	p	+	+	+	+	+	p	p	p	+	+	+	+	10	(71)	4	(29)	0	(0)	0	(0)
21				Generalisability	Discuss the generalisability (external validity) of the study results.	+	-	+	+	+	+	+	-	-	-	-	-	+	+	8	(57)	0	(0)	6	(43)	0	(0)
Other information																											
22				Funding	Give the source of funding and the role of the funders for the present study and, if applicable, for the original study on which the present article is based.	+	-	+	p	+	p	p	-	+	p	p	+	+	p	6	(43)	6	(43)	2	(14)	0	(0)
22		22.1		Accessibility of protocol, raw data, and programming code	Authors should provide information on how to access any supplemental information such as the study protocol, raw data, or programming code.	-	-	-	-	-	-	-	p	-	-	-	p	p	-	0	(0)	3	(21)	11	(79)	0	(0)

Items Nr: 1.3, 4.a, 4.b, 6(a), 6(b), 6.1, 6.2, 6.3, 6.1.a, 7, 7.1.c, 7.1.d, 7.1.e, 7.1.f, 7.1.g, 8, 8.a, 10, 11, 12(b), 12(c), 12(d), 12.1.a, 12.1.b, 12.2, 12.3, 13(a), 13(b), 13.1, 14(a), 14(b), 14(c), 16(a), 16(b), 16(c), 20.a of the three checklists are missing as rated to be out of scope for this review by the study authors.
